# Adipose tissue inflammation mediated by CCL19 overexpression exacerbates experimental periodontitis via elevated circulating saturated fatty acids and osteopontin in Western-diet-fed mice

**DOI:** 10.3389/fimmu.2026.1787572

**Published:** 2026-05-01

**Authors:** Naoaki Ryo, Takanori Shinjo, Miyu Shida, Kohei Sato, Honoka Otsuka, Gulinigeer Dilimulati, Tatsuro Zeze, Yuki Nishimura, Mio Imagawa, Al-kafee Ahmed, Chikako Hayashi, Akiko Yamashita, Takao Fukuda, Terukazu Sanui, Misaki Iwashita, Fusanori Nishimura

**Affiliations:** 1 Department of Periodontology, Faculty of Dental Science, Kyushu University, Fukuoka, Japan; 2 Oral Health/Brain Health/Total Health Research Center, Faculty of Dental Science, Kyushu University, Fukuoka, Japan; 3 Department of Periodontology and Endodontology, Nagasaki University Graduate School of Biomedical Sciences, Nagasaki, Japan

**Keywords:** adipose tissue inflammation, CCL19, free fatty acids, obesity, osteoclastogenesis, osteopontin, periodontitis

## Abstract

**Introduction:**

Individuals with obesity may be at a higher risk of developing severe periodontitis. We previously reported that C-C motif ligand 19 (CCL19) plays a pivotal role in adipose inflammation in obesity and that adipocyte-specific *Ccl19* knock-in (CCL19-KI) mice exhibited greater inflammatory cell infiltration in visceral fat, increased weight gain, and abnormal glucose metabolism than wild-type (WT) mice under a 40% high-fat diet (HFD). In this study, we examined the susceptibility of mice fed diets with different fat contents to the severity of experimental periodontitis.

**Methods:**

Six-week-old male WT and CCL19-KI mice were fed either a normal diet (ND), 40% HFD, or 60% HFD for 8 weeks, after which ligature-induced periodontitis (LIP) was established. Two weeks after ligation, alveolar bone resorption, gingival inflammatory and osteoclastogenic gene expression, and serum free fatty acid (FFA) levels were compared among the groups. Additionally, RNA-sequencing (RNA-seq) was performed on epididymal white adipose tissue (eWAT) of each ligatured mouse to explore potential factors mediating periodontitis aggravation. Furthermore, *in vitro* studies using macrophages were conducted to investigate a possible mechanism in the progression of periodontitis.

**Results:**

Alveolar bone resorption, gingival inflammatory and osteoclastogenic gene expression, and serum FFA levels were significantly higher in ligated CCL19-KI mice than those in ligated WT mice under a 40% HFD. RNA-seq revealed that the OPN gene (*Spp1*) expression in eWAT was significantly upregulated in CCL19-KI mice fed an ND or 40% HFD compared with that in WT mice, which was consistent with serum OPN levels. Peritoneal macrophages from 40% HFD-fed CCL19-KI mice secreted significantly higher amounts of TNF-α than those from WT mice fed the same diet under *Escherichia coli* lipopolysaccharide (LPS) stimulation. Both palmitic acid priming and OPN significantly promoted osteoclastogenesis in RANKL-treated BMMs, and their combination further enhanced this effect.

**Conclusions:**

CCL19-modified adipose tissue inflammation may contribute to the severity of periodontitis via the upregulation of circulating saturated fatty acids and *Spp1*, resulting in enhanced TNF-α production in macrophages and increased osteoclastogenesis in BMMs under mild to moderate obesity.

## 1 Introduction

The global prevalence of obesity has been increasing over the past 30 years (1). Recently, developing countries, in addition to developed countries, have faced an increase in the number of individuals with obesity (2, 3). Westernization of dietary and social lifestyles has led to excess caloric intake and a lack of exercise, which has resulted in an increase in the number of people with obesity and metabolic syndromes (4). Obesity is recognized as a major risk factor for lifestyle-related diseases such as diabetes and hypertension. Therefore, the rising prevalence of obesity is a serious concern, as it leads to escalating medical expenditures and poses a threat to social and economic stability as well as public health.

Obesity is accompanied by the accumulation of excess lipids in adipose tissue as well as dysregulation in metabolism, hemodynamics, and immune function (5–7). Adipose tissue primarily functions as an energy storage site in the body, as well as an endocrine organ that secretes a variety of adipokines. In obesity, large amounts of adipokines, such as pro-inflammatory cytokines and chemokines, are secreted from mature adipose tissues, leading to low-grade systemic inflammation, termed micro-inflammation (8). Micro-inflammation contributes to a marked increase in the risk of developing lifestyle diseases via insulin resistance and abnormalities in glucose and lipid metabolism (9). Recent studies have suggested that obesity not only increases the risk of atherosclerosis and cardiovascular diseases but is also associated with the progression of osteoporosis and periodontitis through disruption of immune response and bone remodeling (10, 11). Therefore, obesity may influence numerous systemic diseases through microinflammation.

Amplification of chronic inflammation in mature adipose tissue is mediated by interactions between adipocytes and immune cells, including macrophages, dendritic cells, and adaptive immune cells (12–14). We previously found that the chemokine C-C motif ligand 19 (CCL19)–C-C motif receptor 7 (CCR7) axis plays a pivotal role in adipose inflammation and adiposity (13). Additionally, we generated mice overexpressing CCL19 in adipocytes (Ccl19 knock-in; CCL19-KI) and demonstrated that activation of the CCL19–CCR7 axis in white adipose tissue accelerated glucose intolerance, insulin resistance, and dyslipidemia, accompanied by the promotion of obesity, especially under a 40% high-fat diet (HFD) (15). Notably, when CCL19-KI and wild-type (WT) mice were fed a 60% HFD, the effects of CCL19 overexpression were completely masked by inflammation caused by marked obesity.

Epidemiological studies have revealed that the Asian population exhibit a unique phenotype, so-called ‘thin-fat’, characterized by adipose tissue maturation for a lower BMI than the Caucasians (16, 17). Obesity has been reported to be associated with the development and severity of periodontitis (11). However, the role of adipose inflammation in modulating susceptibility to periodontitis in mild-to-severe obesity, especially the impact of the maturity of visceral adipose tissue on periodontitis under mild-to-moderate obesity, remains unclear. In this study, we investigated whether the extent of adipose inflammation promotes the progression of periodontitis under mild obesity using CCL19-KI mice and identified potential contributing factors.

## 2 Materials and methods

### 2.1 Mice

CCL19-KI mice were generated as previously described (15). Briefly, the murine *Ccl19* gene was knocked into the *Adipoq* gene locus by CRISPR/Cas9-mediated homologous recombination in mouse zygotes without disrupting the endogenous *Adipoq* gene at the Laboratory Animal Resource Center, University of Tsukuba. Founder (F0) mice were crossed with wild-type C57BL/6J mice to generate F1 mice. Male and female homozygous CCL19-KI mice were mated to maintain the mouse line. For WT mice, male and female C57BL/6 mice were mated to obtain WT mice for the experiments. The latest ARRIVE 2.0 guidelines for animal experiments were followed. All animal experiments and euthanasia protocols were approved by the Institutional Animal Care and Use Committee of Kyushu University (A25–068 and A23-010). WT and CCL19-KI mice were cohabitated under climate-controlled conditions with a 12-hour light/dark cycle and ad libitum access to water and food.

### 2.2 Animal study

**Figure 1:**
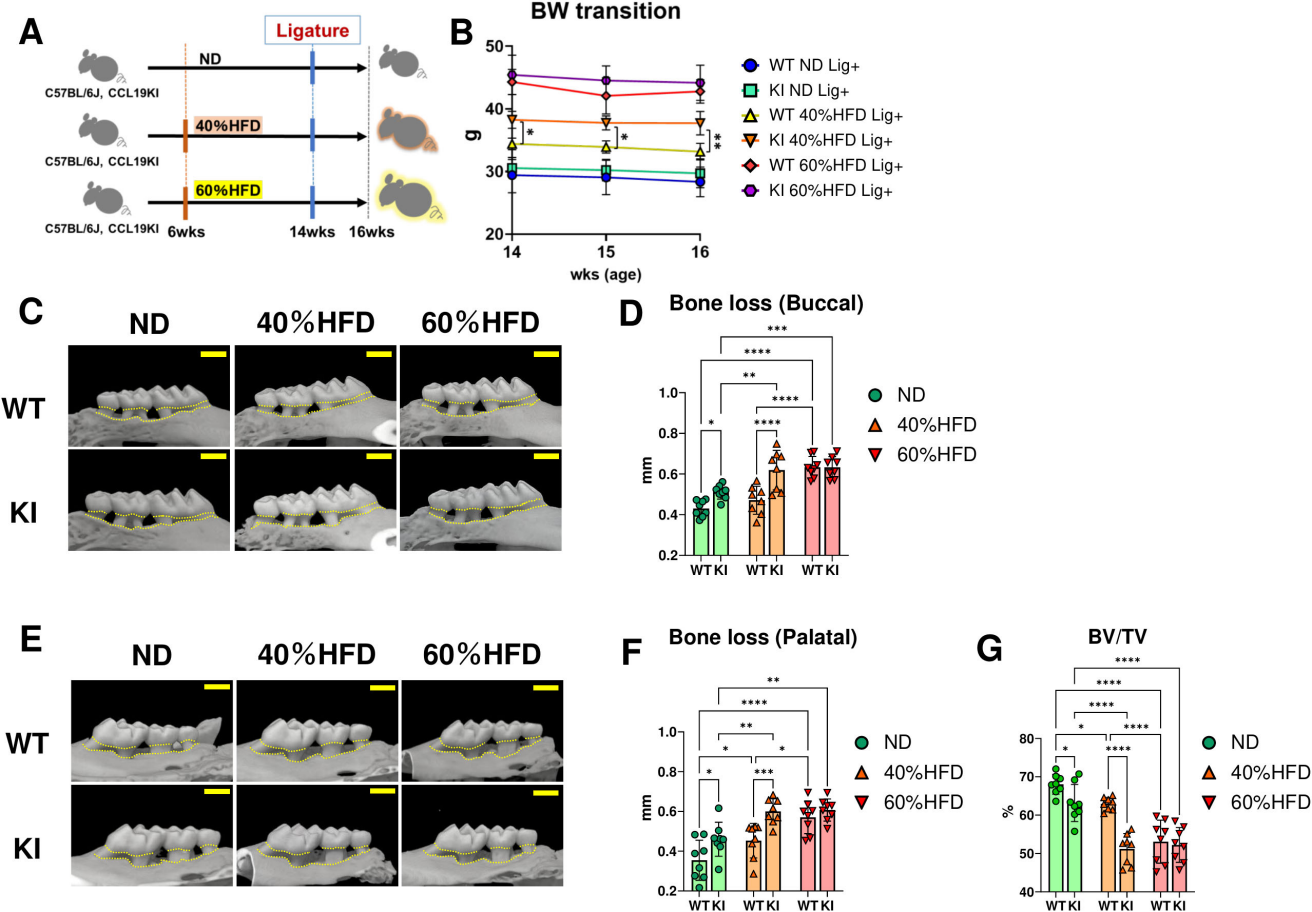
Assessment of alveolar bone loss induced by ligature-induced periodontitis in WT and CCL19-KI mice fed with ND, or 40% or 60% HFD. **(A)** A schema of the *in vivo* study. **(B)** Body weight (BW) transition of each mouse group during LIP (n = 6). WT: wild-type, KI: CCL19-knock-in (KI). The representative images of the maxilla of each mouse and plots of the alveolar bone loss calculated based on the distance from the cemento-enamel junction (CEJ) to the alveolar bone crest (ABC) after 2 weeks of LIP in buccal **(C, D)** and palatal **(E, F)** sides (n = 8). **(G)** Quantified bone volume (BV)-to-tissue volume (TV) ratio in each mouse group after 2 weeks of LIP (n = 8). Scale bar = 1 mm. *p < 0.05, **p< 0.01, ***p< 0.001, ****p< 0.0001. All individual data except for **(B)** are shown in a scatter plot. Data are presented as mean ± SD. P-values were determined by two-way ANOVA followed by *post hoc* tests.

Male CCL19-KI and WT mice were fed with a normal diet (ND; 13.6% fat content) (CRF-1, Oriental Yeast, Tokyo, Japan) from the age of 4 weeks. At 6 weeks old, male CCL19-KI and WT mice were randomly divided into the groups with ND, or 40% fat diet (HFD-40, Oriental Yeast), or 60% fat diet (HFD-60, Oriental Yeast). At 14 weeks old (after 8 weeks of feeding each diet), the mice in each group were further randomly divided into the subgroup with or without ligature-induced experimental periodontitis (LIP) (**Figure 1A**). All of the mice were euthanized at 16 weeks with inhalation anesthesia of sevoflurane and following intraperitoneal injection of a mixed anesthetic (0.3 mg/kg medetomidine (Kyoritsu Pharmaceutical, Tokyo, Japan), 4.0 mg/kg midazolam (SANDOZ, Tokyo, Japan), and 5.0 mg/kg (Meiji Seika Pharma, Tokyo, Japan)) and then collected cardiac blood and perfusion with phosphate-buffered saline (PBS). Body weight in each mouse was monitored once a week.

### 2.3 Induction of ligature-induced experimental periodontitis

Male WT and CCL19-KI mice at 14 weeks old were subject to the induction of LIP as previously reported (18, 19). Briefly, the mice were injected intraperitoneally with a mixed anesthetic containing 0.3 mg/kg medetomidine, 4.0 mg/kg midazolam, and 5.0 mg/kg using a 1 mL syringe, and their unconsciousness was induced. A 6–0 silk thread (Akiyama Medical, Tokyo, Japan) was ligatured around the upper second molar on both sides. After 2 weeks from the ligation, the mice were euthanized to collect blood and maxilla samples. The left halved maxilla was fixed with 10% formalin neutral buffer (Fujifilm Wako, Tokyo, Japan) for micro computed tomography (µCT) analysis and subsequent histological analysis, and the gingiva around upper molars was collected from the right halves’ maxilla into TRIzol reagent (Thermo Fisher Scientific, Waltham, MA, USA). Male mice were used for the experiments to avoid the potential effect of the estrous cycle and the difference of the productive systems (20, 21) on the progression of periodontitis.

### 2.4 Assessment of alveolar bone resorption

Alveolar bone resorption in each mouse was measured using μCT (ScanXmate; Comscan, Kanagawa, Japan) and an analytic software TRI/3D-VIE-FCS (Ratoc System Engineering, Tokyo, Japan). In brief, the 3D photos of the fixed left maxilla were taken, then the distances from cement-enamel junction (CEJ) to alveolar bone crest (ABC) of mesial and distal root of the first molar and second molar and root of the third molar in both buccal and palatal sides were measured by Image J (version 1.43; National Institute of Health, Bethesda, MD, USA) as previously described (22, 23) (**Supplementary Figure 1**). Alveolar bone loss was determined by the subtraction of the sum of the distance from CEJ to ABC from the average of WT mice without LIP. The ratio of bone volume (BV) to tissue volume (TV) was measured by TRI/3D-FCS-MPR3D as in a previous study (24).

### 2.5 RNA extraction and quantitative real-time PCR

Total RNA from the gingival samples immersed in the TRIzol reagent was extracted according to the manufacturer’s instructions. For *in vitro* studies, cultured cells were washed with PBS and collected in ISOGEN II (Nippon Gene, Tokyo, Japan). Cellular total RNA was extracted according to the manufacturer’s protocol. RNA concentration was determined by NanoDrop LITE (Thermo Fisher Scientific). The cDNA was synthesized using Prime Script RT Master Mix (Takarabio, Otsu, Japan). Quantitative reverse transcription-polymerase chain reaction (qRT-PCR) was performed using Luna Universal qPCR Master Mix (New England BioLabs, Ipswich, MA, USA) and StepOne Plus™ Real-Time System (Applied Biosystems, Carlsbad, CA, USA) under the following conditions: 95 °C, 3 min, 95 °C, 3 sec, 60 °C, 30 sec for 40 cycles. Results were recorded and analyzed using StepOne™ software V2.2.2 (Applied Biosystems), utilizing automatically calculated threshold cycles. The ΔΔCT method was used to calculate relative expression levels of individual genes, and 18s rRNA was used as an internal control. The mRNA expression levels in each ligatured mouse were shown as a relative fold of that in WT mice without ligatures. The primer sequences were listed in **Supplementary Table 1**.

### 2.6 Histological section

The left halves of the maxilla and epididymal white adipose tissues (eWAT) fixed in 10% phosphate-buffered formalin for 24 hours were washed with PBS, and then immersed in Osteosoft^®^ (Merck, Darmstadt, Germany) for 48 hours for decalcification. Tissue sections were prepared by embedding demineralized maxillary bone in paraffin and sectioning at a thickness of 5 µm.

### 2.7 Tartrate-resistant acid phosphatase staining

Each tissue section was stained with tartrate-resistant acid phosphatase (TRAP) using the TRAP/ALP Stain Kit (Fujifilm Wako) according to the manufacturer’s instructions. TRAP-positive cells with two or more nuclei formed on the alveolar bone surface of molars were counted as active osteoclasts. For cellular TRAP staining, RANKL-treated mouse bone marrow-derived macrophages were stained with the TRAP/ALP Stain kit. TRAP-positive cells with more than 2 nuclei were counted as osteoclasts. Magnified images were taken using a BZ-X800 microscope (Keyence, Osaka, Japan).

### 2.8 Immunohistochemistry staining

Each tissue section was deparaffinized with xylene and rehydrated with ethanol. Antigen activation was performed at 95 °C for 10 minutes using citrate buffer (pH 6). Sections were washed with PBS for 5 minutes and then incubated with1% BSA for 30 minutes at room temperature for blocking. Sections were stained for CCR7, CCL19, and OPN using anti-rabbit CCR7 antibody (BOSTER, Pleasanton, CA), anti-CCL19 antibody (Abcam, Cambridge, United Kingdom), and anti-OPN antibody (Cell Signaling Technology, Danvers, MA, USA) at a 1:200, 1:1000, and 1:200 dilution with VECTASTAIN ABC Rabbit IgG Kit (Vector Laboratories, Newark, CA, USA) according to the manufacturer’s protocol, respectively.

### 2.9 Serum free fatty acid measurements

Collected cardiac blood was centrifuged at 2000 rpm for 20 minutes at 4 °C to isolate serum. Serum free fatty acid levels were determined by using Free Fatty Acid (FFA) Assays (Cell Biolabs, San Diego, CA, USA) according to the manufacturer’s instructions.

### 2.10 RNA-sequence

#### 2.10.1 Sample preparation

The eWAT from each ligatured mouse was harvested. After removal of lymph nodes, the total RNA was extracted using PureLink^®^ RNA Mini kit with PureLink™ DNase Set (Thermo Fischer Scientific) according to the manufacturer’s guidance. RNA samples were quantified by an ND-1000 spectrophotometer (NanoDrop Technologies, Wilmington, DE, USA), and the quality was confirmed with a Tapestation (Agilent Technologies, Santa Clara, CA, USA).

#### 2.10.2 RNA-sequencing

The sequencing libraries were prepared from 200 ng of total RNA with the MGIEasy rRNA Depletion kit and MGIEasy RNA Directional Library Prep Set (MGI Tech, Shenzhen, Guangdong, China) according to the manufacturer’s instructions. The libraries were sequenced on the DNBSEQ-G400 FAST Sequencer (MGI Tech) with a pair-end 150 nt strategy.

#### 2.10.3 Sequencing data analysis

All sequencing reads were trimmed of low-quality bases and adapters with Trimmomatic (v.038) (25). Trimmed reads were mapped to the transcript in the reference mouse mm10 using the Bowtie2 aligner within RSEM (26, 27). The abundance estimation of genes and isoforms with RSEM generated basic count data. We used the edgeR program to detect differentially expressed genes (DEGs). Normalized counts per million (CPM) values, log fold-changes (LogFC), and p-values were obtained from the gene-level raw counts. Then, we established criteria for DEGs: p-value ≤ 0.05 and ratio ≥ 1.5-fold (up-regulated genes) or 0.67 (down-regulated genes). Gene Ontology (GO) pathway analysis was performed using Functional Annotation Bioinformatics Microarray Analysis (DAVID) software (https://davidbioinformatics.nih.gov).

### 2.11 Peritoneal macrophage collection

Peritoneal macrophages were isolated from each mouse group as previously reported (19). Briefly, WT and CCL19-KI mice fed with ND or 40% HFD were intraperitoneally injected with 2% thioglycolate (Sigma-Aldrich, Saint Louis, MO, USA) broth in PBS. After 2 days of injection, the cervical vertebrae of the mice were dissected under anesthesia with inhalation of sevoflurane, and the mice were euthanized. The abdominal skin was removed using forceps to expose the peritoneum. Ice-cold PBS (5 mL) was intraperitoneally injected into the peritoneal cavity, then the abdomen of the mouse was gently massaged for 2 min, and PBS containing macrophages was collected using a 5 mL syringe. After centrifugation at 4 °C at 1000 rpm for 5 min, the pelleted cells were resuspended in 5 mL of complete RPMI-1640 (Nacalai Tesque, Kyoto, Japan) with 10% FBS supplemented with penicillin/streptomycin mixed solution (PC/SM; Nacalai Tesque). Then, cells were cultured in a 6 cm dish or 12-well plates. After overnight (~12 hours) incubation, the culture medium was exchanged to remove non-adhered cells. Adherent cells were used for further experiments.

The purity of peritoneal macrophages was confirmed by flow cytometry as follows (**Supplementary Figure 2**). Cultured peritoneal macrophages were collected in PBS using a cell scraper. Single-cell suspensions were washed with PBS and stained with live/dead fixable viability stain (Thermo Fisher Scientific). Fc receptors were blocked using TruStain FcX™ PLUS (anti-mouse CD16/32 antibody, Biolegend, San Diego, CA, USA) before surface staining with antibodies of interest (**Supplementary Table 2**) in FACS wash buffer (Biolegend, 10 min, 4 °C). Isotype controls were used to confirm antibody specificity. The cells were incubated in the dark for 30 min at 4 °C and analyzed using a BD FACSLyric flow cytometer (BD Biosciences). Data were processed using BD FACSuite™ software v1.6 (BD Biosciences).

### 2.12 Immunofluorescent stain

Each paraffin-embedded eWAT sections was deparaffinized as mentioned above. After antigen retrieval and blocking, the sections were incubated with primary antibodies (anti-rabbit F4/80 antibody (Abcam) at a 1:200 and anti-OPN antibody (R&D Systems, Minneapolis, MN, USA) at a 1:20 Dilution) at 4°C overnight in the shade. The sections were then washed with OBS and incubated with secondary antibodies (anti-rabbit IgG with AF488 (Abcam) and anti-goat IgG with AF647 (Abcam)) for 2 hours at room temperature in the shade. Nuclei were stained with Slow Fade Diamond Antifade Mountant with DAPI (Invitrogen, Waltham, MA, USA). Visualization was performed using a Keyence BZ-X800 fluorescence microscope. Positively stained cells in the eWAT were quantified using ImageJ software.

### 2.13 Cell culture

The mouse macrophage cell line RAW264.7 (TIB-71™) was obtained from the American Type Culture Collection (ATCC, Manassas, VA, USA). RAW264.7 cells were grown in 10% heat-inactivated fetal bovine serum (FBS, Biowest, Vieux Bourg, France) and PC/SM (Nacalai Tesque) in Dulbecco’s modified Eagle’s medium (DMEM) with 25 mM D-glucose (Nacalai Tesque). No human studies were performed in this study.

### 2.14 *In vitro* study

The 5.0×10^5^ cells of peritoneal macrophages were cultured in a 12-well plate in RPMI with 10% FBS supplemented with PC/SM. Peritoneal macrophages were stimulated with 10 ng/mL *Escherichia coli* (*E. coli*) O111:B4 LPS (Sigma-Aldrich) after medium change. The culture media and cellular RNA were collected and extracted after 24 or 6 hours of *E. coli* LPS stimulation for ELISA or qPCR, respectively. RAW264.7 cells were cultured in a 12-well plate at 2.5×10^5^ cells under DMEM-HG with 10% FBS supplemented with PC/SM. Palmitate was prepared according to a previous report (28). In brief, palmitic acid (Fujifilm Wako) was dissolved in 100% ethanol to 200 mmol/l at 70 °C. Then, the palmitic acid solution was diluted to 5 mmol/L by adding a 10% solution of fatty acid-free Bovine Serum (BSA) with incubation at 55 °C for 10 min. The complex solution was allowed to cool to room temperature and filtered through a 0.45-μm pore membrane filter. The stock solution (5 mmol/L) was warmed prior to use, and the cells were preincubated with a final 200 µmol/L of palmitate for 24 hours. Then, the medium was changed into fresh media, and RAW264.7 cells were stimulated with *E. coli* LPS for 6 and 24 hours to measure gene expression or cytokine production, respectively. An equal amount of BSA (200 µmol/L) was used for the control. For RAW264.7 cells, cellular protein was collected in CytoBuster Protein Extraction Buffer (Merck, Darmstadt, Germany) and measured using the Quick Start Bradford Protein Assay Kit (Bio-Rad Laboratories) according to the manufacturer’s protocol. RAW264.7 cells were stimulated with 0.1 to 10 ng/mL of rmCCL19 (Biolegend) for 2, 6, and 12 hours to measure gene expression of inflammatory genes.

### 2.15 Enzyme-linked immunosorbent assay

*E. coli* LPS-stimulated secretion of TNFα from peritoneal macrophages isolated from WT and CCL19-KI mice with each diet and RAW264.7 cells co-stimulated with 200 µM of palmitate, or BSA was measured using ELISA MAX™ Deluxe Set Mouse TNF-α (Biolegend). Absorbance was measured at 450 nm using a microplate reader (Bio-Rad Laboratories, Hercules, CA, USA). For quantification, the secreted amount of TNFα was calculated by normalization of the total TNFα amount of the cultured medium with cellular protein amount, since the proliferative ratio of RAW264.7 cells is fast (around 12~16 hours), and palmitate and *E. coli* LPS may affect the proliferative activity (29). Serum Osteopontin levels in each mouse were determined using the Quantikine ELISA kit (R&D Systems).

### 2.16 Bone marrow-derived macrophages culture

Bone marrow-derived macrophages (BMMs) were obtained as previously reported (30). Briefly, femur and tibia were collected from six to eight-week-old male C57BL/6 mice and the adhered tissues. The bone ends were cut off using scissors, and the marrow cavity was flushed with α-MEM by slowly injecting from one end of the bone using a sterile needle to collect bone marrow into the 1.5 mL tube. Bone marrow cells were then washed with α-MEM, and erythrocytes were removed by incubation in ACK Lysis buffer (Gibco, Waltham, MA) at 37 °C for 5 minutes. After washing, cells were cultured in α-MEM containing 10% FBS, PC/SM, and recombinant mouse (rm) M-CSF (100 ng/mL; Biolegend) at 5×10^6^ cells in a 10 cm culture dish. After 3 days of culture, cells were washed vigorously with PBS twice to remove nonadherent cells, and incubated with 2.5mL of 0.02% EDTA in PBS for 5 minutes, and suspended by pipetting using a 5 mL pipette until detaching cells from the dish. After washing with α-MEM with 10% FBS, cells were spun down and resuspended in fresh α-MEM with 10% FBS. BMMs were then seeded in a 48-well plate for osteoclast differentiation and pit assay (with 4×10^4^ cells/mL).

### 2.17 Induction and assessment of osteoclastic differentiation and activity

BMMs were pretreated with palmitate or BSA (200 µM, respectively) for 24 hours, then the culture media was changed. rmRANKL (R&D Systems; 25 ng/mL) was added to the BMMs in the presence or absence of rmOsteopontin (100 ng/mL; Biolegend) for 5 days (with medium change at day 3). For the OPN neutralization study, BMMs were incubated with anti-OPN antibody (R&D systems) at a 1:100 dilution from the pretreatment with palmitate or BSA to 5 days after induction of differentiation.

The TRAP/ALP Stain kit was used for assessment of osteoclast formation as described above. For assessment of osteoclast formation, TRAP-positive cells with equal to or more than 7 nuclei were counted as previously reported (31). The area of each osteoclast with ≦ 7 nuclei was measured to generate a histogram of the osteoclast area. For analysis of osteoclast activity, a bone resorption (pit) assay plate 48 or 96 (PG Research, Tokyo, Japan) coated with calcium phosphate was used according to the manufacturer’s instructions. Briefly, after 5 or 10 days of RANKL treatment, BMMs were lysed with 5% sodium hypochlorite (Sigma-Aldrich) for 5 minutes, and the photos were taken using the microscope BZ-1000. The resorption area was measured using ImageJ.

### 2.18 Statistical analysis

All data are presented as mean ± standard deviation (SD). All comparisons were two-way analyses of variance performed for multiple groups using GraphPad Prism10 software (GraphPad Software, San Diego, CA). Statistical significance was set at p < 0.05.

## 3 Results

### 3.1 LIP-mediated alveolar bone resorption in CCL19-KI mice is enhanced compared with that in WT mice under a 40% HFD

Body weight of both WT and CCL19-KI mice increased in proportion to the fat content of the diet (**Supplementary Figure 3**). CCL19-KI mice showed significantly higher body weight than WT mice under 40% HFD feeding from 12 to 14 weeks of age (**Supplementary Figure 3**) and during LIP (**Figure 1B**). In WT mice, LIP-mediated alveolar bone resorption under a 40% HFD was comparable to that under a normal diet (ND), whereas resorption under a 60% HFD was significantly greater than that under an ND or 40% HFD (**Figures 1C–F**). In CCL19-KI mice, LIP-induced alveolar bone loss under both 40% and 60% HFD was significantly greater than that under an ND, and alveolar bone loss induced by LIP under a 40% HFD was comparable to that under a 60% HFD (**Figures 1C–F**). Notably, CCL19-KI mice displayed a significant increase in LIP-mediated alveolar bone resorption compared to WT mice under an ND or 40% HFD (**Figures 1C–F**). No significant differences in alveolar bone loss were observed among mice without ligatures (**Supplementary Figure 4**). WT mice exhibited a proportional LIP-mediated decrease in the BV/TV ratio based on the dietary fat content, whereas CCL19-KI mice showed comparable BV/TV ratios under 40% and 60% HFDs, both significantly lower than that under an ND (**Figure 1G**). Notably, CCL19-KI mice showed a significant LIP-mediated decrease in the BV/TV ratio under an ND or 40% HFD but not under a 60% HFD (**Figure 1G**).

### 3.2 LIP-mediated gene expression of inflammation and osteoclastogenesis in the gingiva of CCL19-KI mice is higher than that in WT mice under a 40% HFD

**Figure 2:**
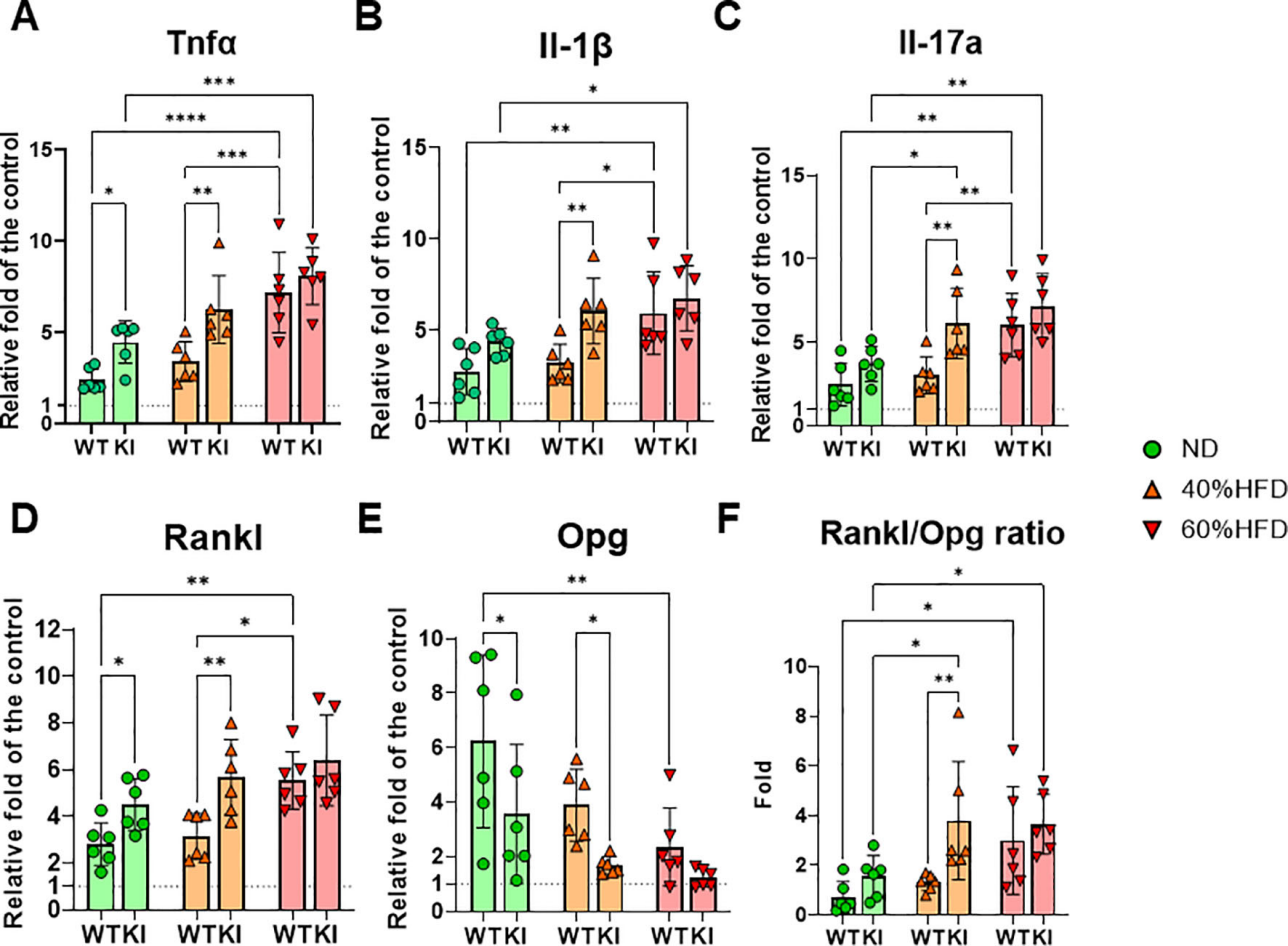
Inflammatory and osteoclastogenesis-related gene expression in the gingiva of each mouse group. Relative mRNA expression of **(A)** Tnfα, **(B)** Il-1β, **(C)** Il-17a, **(D)** Rankl, and **(E)** Opg expression in the gingiva of each ligatured mouse group (n = 6). All of the data were normalized to the mean expression in the gingiva of WT mice without ligatures. **(F)** The ratio of Rankl to Opg mRNA expression in the gingiva of each ligatured mouse group (n = 6). All of the data were shown as the relative fold to the mean of ND-fed mice without ligatures. *p < 0.05, **p< 0.01, ***p< 0.001, ****p< 0.0001. All individual data are shown in a scatter plot. Data are presented as mean ± SD. P-values were determined by two-way ANOVA followed by *post hoc* tests.

To assess inflammation- and osteoclastogenesis-related gene expression in the gingiva of each ligatured mouse group, qPCR was performed. In WT mice, LIP-mediated gingival expression of *Tnfα*, *Il1β*, *Il17a*, and *Rankl* under a 60% HFD was significantly upregulated compared with that under ND or 40% HFD (**Figures 2A–D**). Osteoprotegerin (*Opg*) expression in the gingiva of WT mice with ligatures fed a 60% HFD was significantly downregulated compared with that in ND-fed WT mice with ligatures (**Figure 2E**). In CCL19-KI mice, ligature-induced gingival expression of *Tnfα*, *Il1β*, and *Il17a* under a 60% HFD was significantly increased compared with that under ND (**Figures 2A–D**). Ligatured WT and CCL19-KI mice fed a 60% HFD displayed a higher ratio of *Rankl* to *Opg* expression in the gingiva than ligatured ND-fed WT and CCL19-KI mice (**Figure 2F**).

ND-fed CCL19-KI mice with ligatures exhibited significant upregulation of gingival *Tnfα* and *Rankl* mRNA expression and significant downregulation of gingival *Opg* mRNA expression compared with those of ND-fed WT mice with ligatures (**Figures 2A–E**). Furthermore, CCL19-KI mice with ligatures fed a 40% HFD showed significantly higher gingival expression of *Tnfα*, *Il1β*, *Il17a*, and *Rankl*, and significantly lower gingival expression of *Opg* than those in WT mice with ligatures fed the same diet (**Figures 2A–E**). All inflammatory and osteoclastogenic gene expression in the gingiva of WT mice with ligatures was similar to that of CCL19-KI mice with ligatures under a 60% HFD (**Figures 2A–E**). The ratio of *Rankl* to *Opg* expression in the gingiva of mice with ligatures fed a 40% HFD was significantly higher than that in WT mice with ligatures fed the same diet (**Figure 2F**).

### 3.3 Ligatured CCL19-KI mice exhibit a significant increase in osteoclasts surrounding alveolar bone compared with ligatured WT mice under an ND or 40% HFD

**Figure 3:**
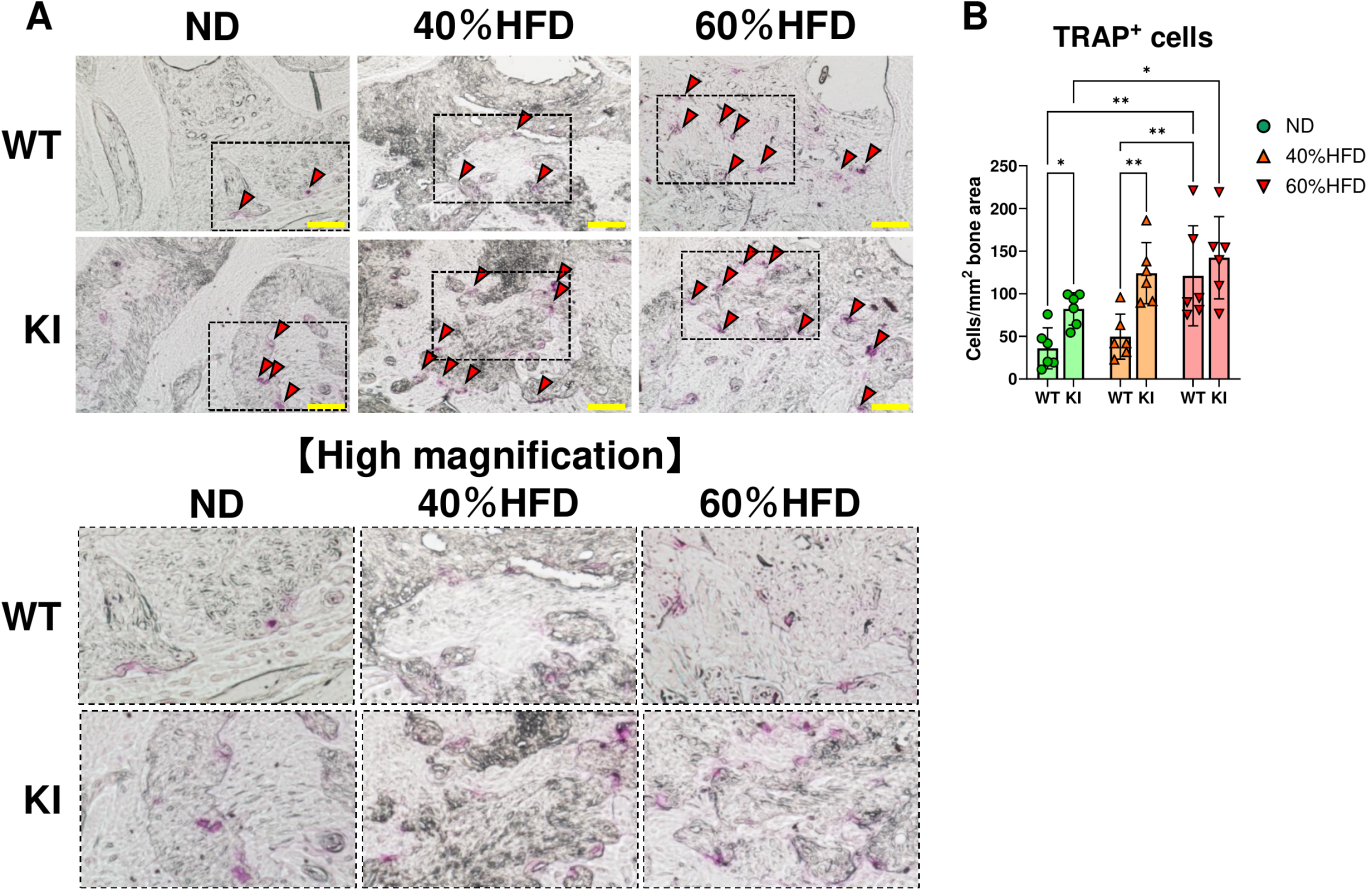
Assessment of osteoclasts on the surface of the alveolar bone of each mouse after 2 weeks of LIP. **(A)** The representative images of TRAP staining in each mouse group. Red arrows indicate TRAP-stained cells. Magnified photos corresponding to the dashed rectangles are shown in each lower image. Scale bar = 100 μm. **(B)** The quantified plots of TRAP-positive cells in each mouse group (n = 6). *p < 0.05, **p< 0.01. All individual data are shown in a scatter plot. Data are presented as mean ± SD. P-values were determined by two-way ANOVA followed by *post hoc* tests.

TRAP staining was performed to detect osteoclasts surrounding alveolar bone in WT and CCL19-KI mice with or without ligatures. No significant differences in the number of TRAP-positive cells were observed among WT and CCL19-KI mice without ligatures, regardless of diet (**Supplementary Figure 5**). In both WT and CCL19-KI mice, the number of TRAP-positive cells under an ND was similar to that under a 40% HFD, whereas the number under a 60% HFD was significantly higher than that under an ND or 40% HFD (**Figures 3A, B**). Importantly, the number of TRAP-positive cells in ligatured CCL19-KI mice fed an ND or 40% HFD was significantly higher than that in ligatured WT mice fed the same diets, whereas that in ligatured CCL19-KI mice fed with 60% HFD was comparable to that in ligatured WT mice under a 60% HFD (**Figures 3A, B**). CCL19-KI mice exhibited compatible CCR7 expression levels in periodontal tissue with WT mice under both ligatured and non-ligatured conditions (**Supplementary Figure 6**). Additionally, CCL19-positive cells were not detected in the periodontal tissue regardless of LIP in both mice (**Supplementary Figure 7**), whereas CCL19-expressing cells were detected in the stromal vascular fraction area of eWAT (**Supplementary Figure 8**).

### 3.4 Ligatured CCL19-KI mice show a significant elevation of serum FFA levels compared to those in ligatured WT mice under an ND or 40% HFD

**Figure 4:**
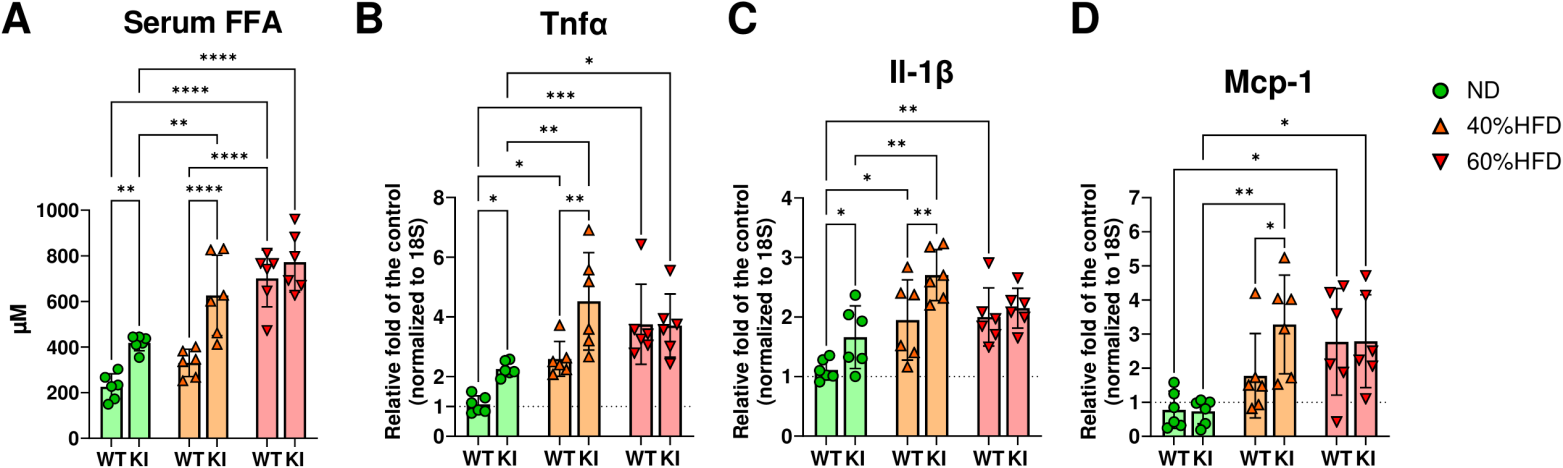
The serum free fatty acid levels and proinflammatory gene expression of eWAT in each ligatured mouse group. **(A)** Serum FFA levels and **(B)** relative *Tnfα*, **(C)** *Il-1β*, and **(D)** *Mcp-1* expression levels in the eWAT of each ligatured mouse (n = 6). All of the qPCR data were shown as the relative fold to the mean of ND-fed mice without ligatures. *p<0.05, **p< 0.01, ***p< 0.001, ****p< 0.0001. All individual data are shown in a scatter plot. Data are presented as mean ± SD. P-values were determined by two-way ANOVA followed by *post hoc* tests.

Next, ELISA was performed to measure the serum FFA levels in each ligatured mouse group, as circulating FFA levels may be associated with the progression of periodontitis (32). The ELISA revealed that serum FFA levels were elevated in proportion to the fat content in the diets of both ligatured WT and CCL19-KI mice (**Figure 4A**). In ligatured WT mice, serum FFA levels under an ND were similar to those under 40% HFD feeding, whereas those under a 60% HFD were significantly and highly elevated compared to the other diet-fed groups (**Figure 4A**). In ligatured CCL19-KI mice, serum FFA levels under a 60% HFD were significantly higher than those under an ND but not significantly different from those under a 40% HFD (**Figure 4A**). Notably, serum FFA levels in ligatured CCL19-KI mice were significantly higher than those in ligatured WT mice (**Figure 4A**).

### 3.5 Ligatured CCL19-KI mice exhibited a significant upregulation of inflammatory gene expressions in eWAT compared to those in ligatured WT mice under a 40% HFD

To assess inflammatory gene expressions in eWAT of each mouse, qPCR was performed. *Tnfα*, *Il-1β*, and *Mcp-1* expression in the eWAT of ligatured WT mice fed a 60% HFD was significantly higher than that in WT mice fed an ND (**Figures 4B–D**). *Il-1β* but not *Tnfα* and *Mcp-1* expression was significantly upregulated in the eWAT of ligatured WT mice fed a 40% HFD compared to ligatured WT mice fed an ND (**Figures 4B–D**). In CCL19-KI mice, *Tnfα*, *Il-1β*, and *Mcp-1* expressions in the eWAT were significantly elevated by 40 and 60% HFD feeding compared to ND feeding (**Figures 4B–D**). These inflammatory gene expressions in the eWAT of ligatured CCL19-KI mice were significantly higher than those of ligatured WT mice under an ND and 40% HFD, whereas those in the eWAT were not significantly different from those of ligatured WT mice under a 60% HFD (**Figures 4B–D**).

### 3.6 Inflammatory responses to LPS are primed and enhanced in macrophages from CCL19-KI mice fed a 40% HFD and further augmented by PA co-stimulation

**Figure 5:**
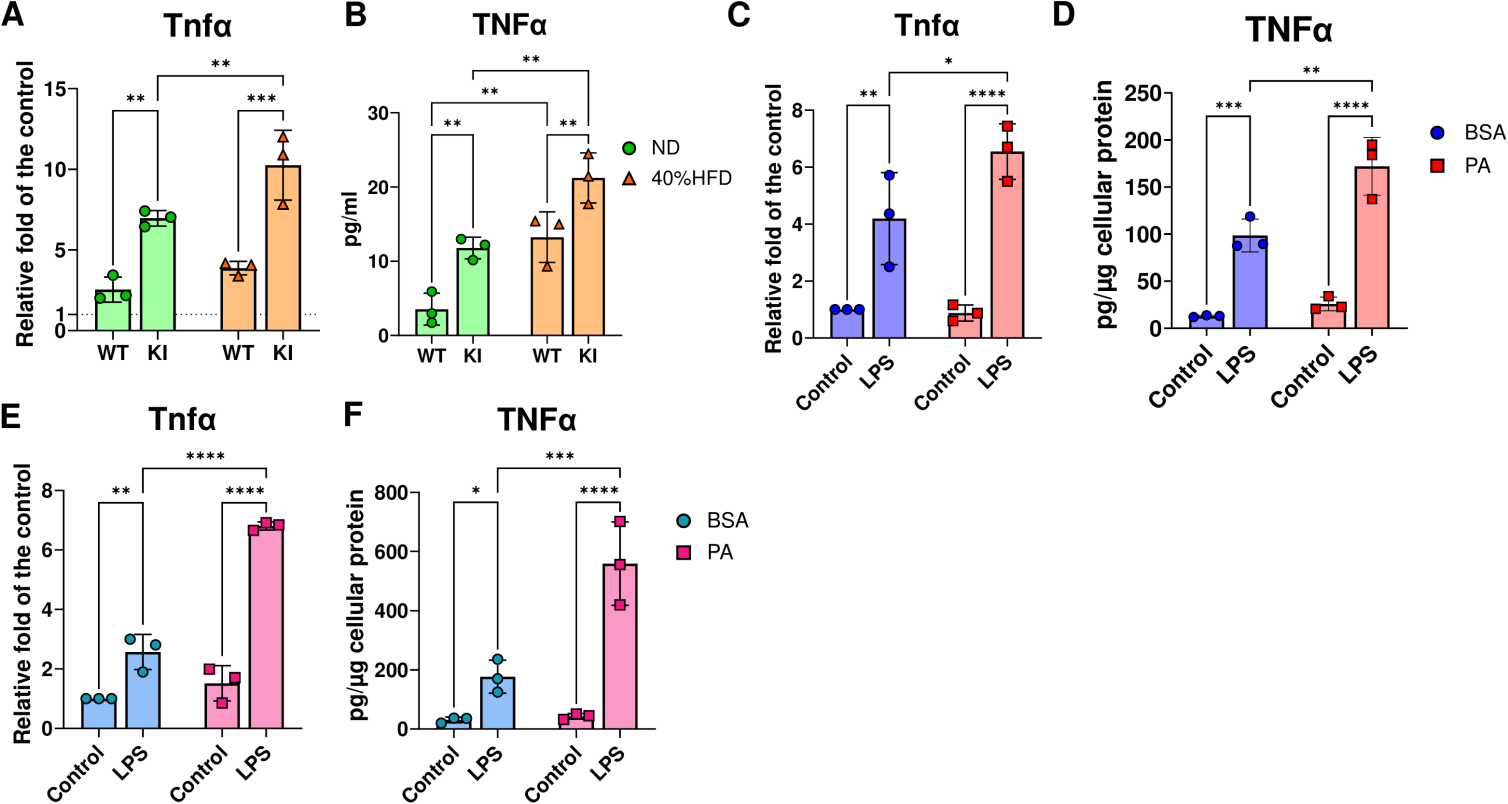
Inflammatory response to E. coli LPS in the peritoneal macrophages from ND or 40% HFD-fed WT mice, RAW 264.7 cells pre- and co-stimulation with palmitate. The mRNA expression **(A)** and secreted protein **(B)** of TNFα in/from peritoneal macrophages obtained from ND or 40% HFD-fed WT mice (n = 3). The mRNA expression **(C)** and secreted protein **(D)** of TNFα from RAW264.7 cells treated with 10 ng/mL E. coli LPS with pretreatment of 200µM of BSA or palmitate (n = 3). The mRNA expression **(E)** and secreted protein **(F)** of TNFα from RAW264.7 cells treated with 10 ng/mL E. coli LPS co-stimulated with 200µM of BSA or palmitate (n = 3). The secreted TNFα amount was normalized with the cellular protein amount were displayed in **(D)** and **(F)** to reflect the potential toxic effect of palmitate. *p < 0.05, **p< 0.01, ***p< 0.001, ****p< 0.0001. All individual data are shown in a scatter plot. Data are presented as mean ± SD. P-values were determined by two-way ANOVA followed by *post hoc* tests.

To confirm whether macrophages from HFD-fed mice with higher serum FFA levels evoked a stronger inflammatory response to infection, which could be associated with greater LIP-mediated alveolar bone loss, peritoneal macrophages from WT and CCL19-KI mice fed ND or 40% HFD were isolated and stimulated with *E. coli* LPS. qPCR analysis showed that LPS-induced *Tnfα* expression in macrophages from CCL19-KI mice was significantly higher, by approximately 1.5-fold, than that in macrophages from WT mice under an ND or 40% HFD (**Figure 5A**). In contrast, 40% HFD did not significantly affect LPS-induced *Tnfα* expression in macrophages from WT mice. The ELISA showed that macrophages from CCL19-KI mice secreted significantly higher amounts of TNF-α than those from WT mice under an ND or 40% HFD (**Figure 5B**). In addition, macrophages from WT and CCL19-KI mice fed a 40% HFD produced higher levels of TNF-α than those from their respective ND-fed counterparts (**Figure 5B**).

PA is one of the most abundant saturated fatty acids in the circulation (33). PA-primed macrophages are more responsive to infectious stimuli, resulting in a pronounced inflammatory response (34). To assess whether PA primes inflammatory responses in macrophages, RAW264.7 cells were incubated with LPS with PA pre-treatment or co-stimulation. qPCR and ELISA analyses showed that LPS-induced gene and protein expression of TNF-α in PA-primed RAW264.7 cells was significantly enhanced compared with that in RAW264.7 cells preincubated with BSA (**Figures 5C, D**). In addition, LPS-induced gene and protein expression of TNF-α in RAW264.7 cells co-stimulated with PA was significantly higher than that in cells co-stimulated with BSA (**Figures 5E, F**), and this increase was greater than that observed in LPS-stimulated RAW264.7 cells subjected to PA pre-treatment (**Figures 5D, F**). Besides, rmCCL19 did not induce inflammatory gene expressions in RAW264.7 cells (**Supplementary Figure 9**).

### 3.7 Ligatured CCL19-KI mice show significantly higher OPN expression in visceral adipose tissue and serum than ligatured WT mice under a 40% HFD

**Figure 6:**
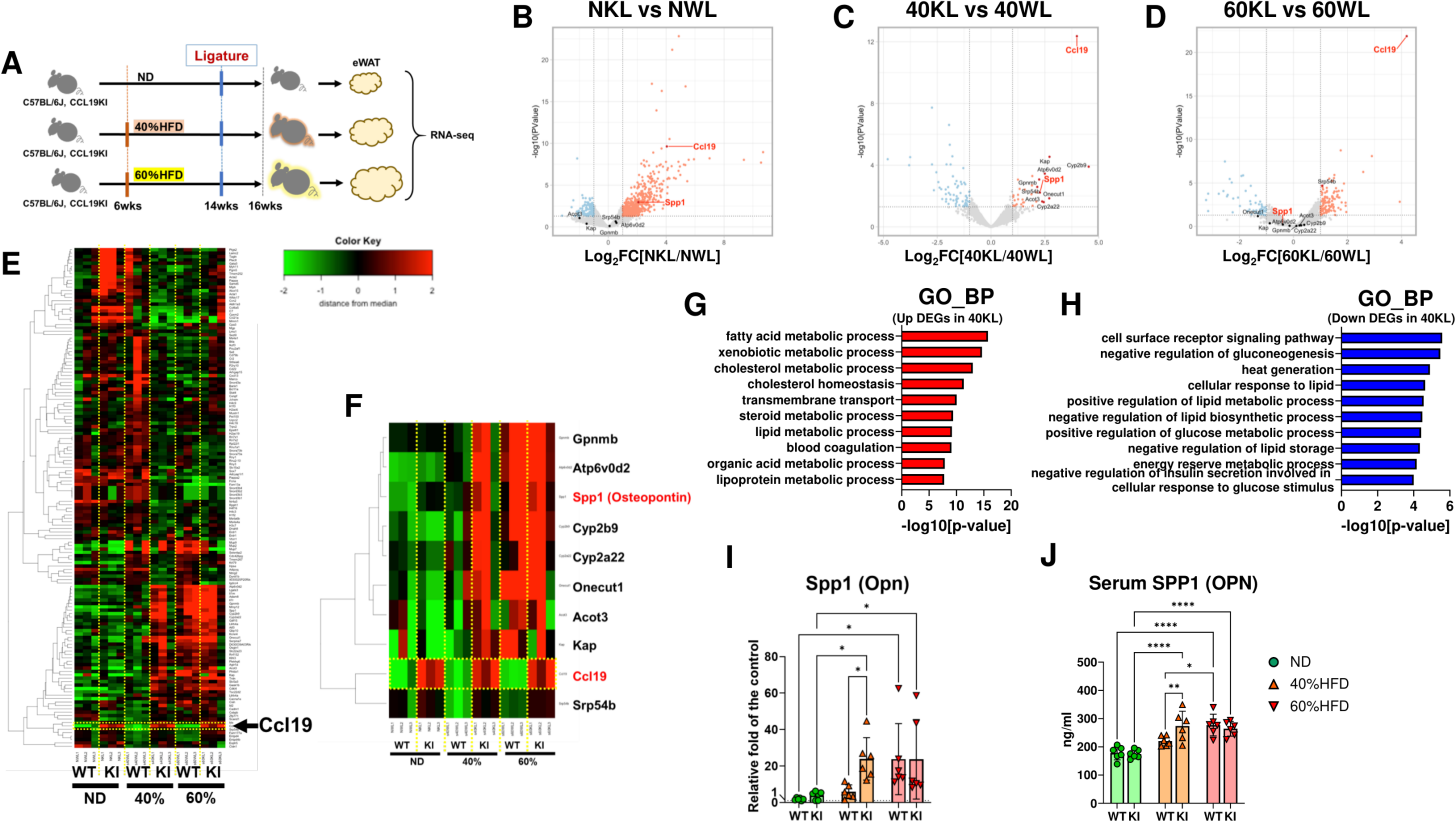
Comprehensive analysis of differentially expressed genes of epididymal white adipose tissue of each ligatured mouse and validation. **(A)** The schematic of the strategy of RNA-seq in the eWAT of each mouse group. The volcano plots of the comparison between ND-fed ligatured WT (NWL) and CCL19-KI (NKL) mice **(B)**, 40% HFD-fed ligatured WT (40WL) and CCL19-KI (40KL) mice **(C)**, and 60% HFD-fed ligatured WT (60WL) and CCL19-KI (60KL) mice **(D)**. **(E)** Heatmap of 145 DEGs in the eWAT of 40KL compared with 40WL. **(F)** Heatmap of the top 10 DEGs in the eWAT of 40KL compared with 40WL. GO analysis in the biological process (BP) of upregulated **(G)** and downregulated DEGs **(H)** in the eWAT of 40KL compared with 40WL. Plots of the relative mRNA expression of Spp1/Opn in the eWAT of each ligatured mouse (n = 6). **(I)** Serum SPP1/OPN levels in each ligatured mouse (n = 6). *p < 0.05, **p< 0.01, ***p< 0.001, ****p< 0.0001. All individual data are shown in a scatter plot **(I, J)**. Data are presented as mean ± SD. P-values were determined by two-way ANOVA followed by *post hoc* tests.

To explore potential factors derived from inflamed adipose tissue that contribute to the progression of LIP-mediated alveolar bone loss in CCL19-KI mice fed a 40% HFD, RNA-seq was performed using eWAT RNA from ligatured mice fed each diet (**Figure 6A**). Volcano plots showed significant upregulation of *Ccl19* expression in the eWAT of CCL19-KI mice compared with that of WT mice under all dietary conditions (**Figures 6B–D**). Among the DEGs identified in the comparison between CCL19-KI and WT mice under a 40% HFD, 145 genes were detected (**Figures 6C, E**). *Gpnmb*, *Atp6v0d2*, *Spp1*, *Cyp2b9*, *Cyp2a22*, *Onecut1*, *Acot3*, *Kap*, *Ccl19*, and *Srp54b* were identified as the top 10 upregulated DEGs in the eWAT of ligatured CCL19-KI mice fed a 40% HFD compared with those of ligatured WT mice fed the same diet (**Figure 6F**). GO analysis revealed that multiple metabolic processes, including fatty acid, cholesterol, and lipid metabolism, were enriched among both upregulated and downregulated DEGs (**Figures 6G, H**).

OPN has been recently shown to regulate osteoclastogenesis in obesity and bone metastasis (35, 36). OPN gene (*Spp1*) expression in the eWAT of ligatured WT mice fed a 60% HFD was significantly higher than that in WT mice fed an ND or 40% HFD (**Figure 6G**), whereas *Spp1* expression in the eWAT was not significantly different from that of ligatured WT mice fed a 40% HFD compared to that in ND-fed ligatured WT mice (**Figure 6I**). ELISA revealed that serum OPN levels increased in proportion to dietary fat content in both WT and CCL19-KI mice with ligatures (**Figure 6J**). Notably, ligatured CCL19-KI mice fed a 40% HFD exhibited significantly higher serum OPN levels than ligatured WT mice fed the same diet (**Figure 6J**). OPN was mainly detected in the periodontal ligament and surface of alveolar bone, but not in epithelial and connective tissue (**Supplementary Figure 10**).

### 3.8 Ligatured CCL19-KI mice displayed significantly larger F4/80 and OPN-positive area in visceral adipose tissue than ligatured WT mice under a 40% HFD

**Figure 7:**
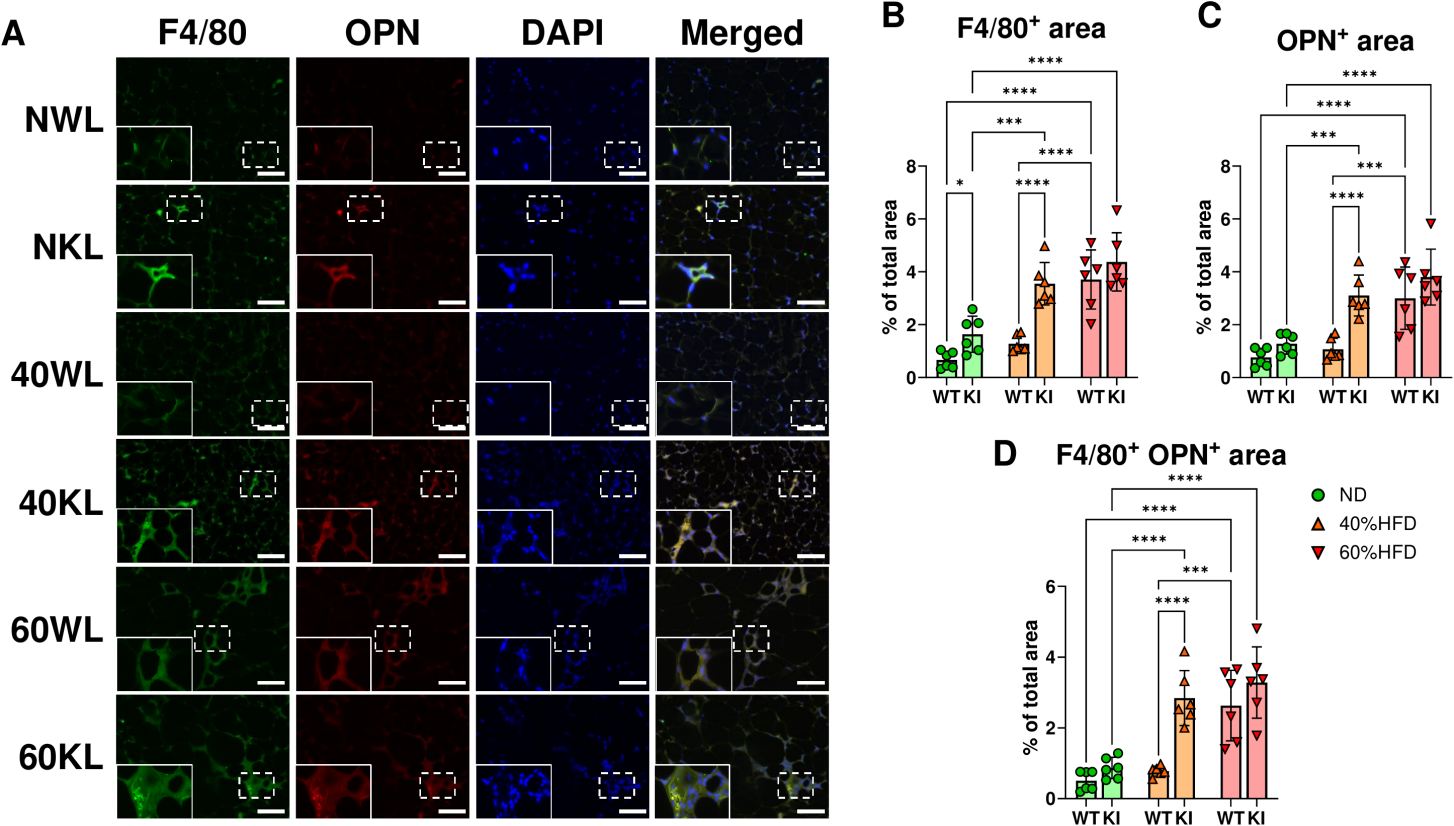
Assessment of the F4/80 and OPN-positive area in eWAT of each ligatured mouse. **(A)** Representative photos of F4/80, OPN, DAPI, and the merged stained area. Magnified photos corresponding to the dashed rectangles are shown at the upper left in each lower image. **(B)** Plots for the quantified F4/80-positive area of each mouse. **(C)** Plots for the quantified OPN-positive area of each mouse. **(D)** Plots for the quantified F4/80 and OPN-double positive area of each mouse. *p < 0.05, **p< 0.01, ***p< 0.001, ****p< 0.0001. All individual data are shown in a scatter plot (n = 6). Data are presented as mean ± SD. P-values were determined by two-way ANOVA followed by *post hoc* tests.

To confirm the source of OPN in the eWAT in response to high-fat diets, immunofluorescent staining was performed. F4/80 and OPN positive area in eWAT of ligatured WT mice with a 60% HFD was significantly increased compared to those with an ND and 40% HFD, whereas those of ligatured CCL19-KI mice with a 40% and 60% HFD were significantly elevated compared to those with an ND (**Figures 7A–C**). Both area in the eWAT of ligatured CCL19-KI mice were significantly larger than those of ligatured WT mice under 40% HFD but not ND and 60% HFD (**Figures 7A–C**). Furthermore, most of the OPN-positive area was colocalized with F4/80 (**Figures 7A, D**), which was consistent with a previous report showing OPN was mainly derived from macrophages in visceral adipose tissue under 60% HFD (35).

### 3.9 PA priming and OPN positively regulate the differentiation and activity of osteoclasts

**Figure 8:**
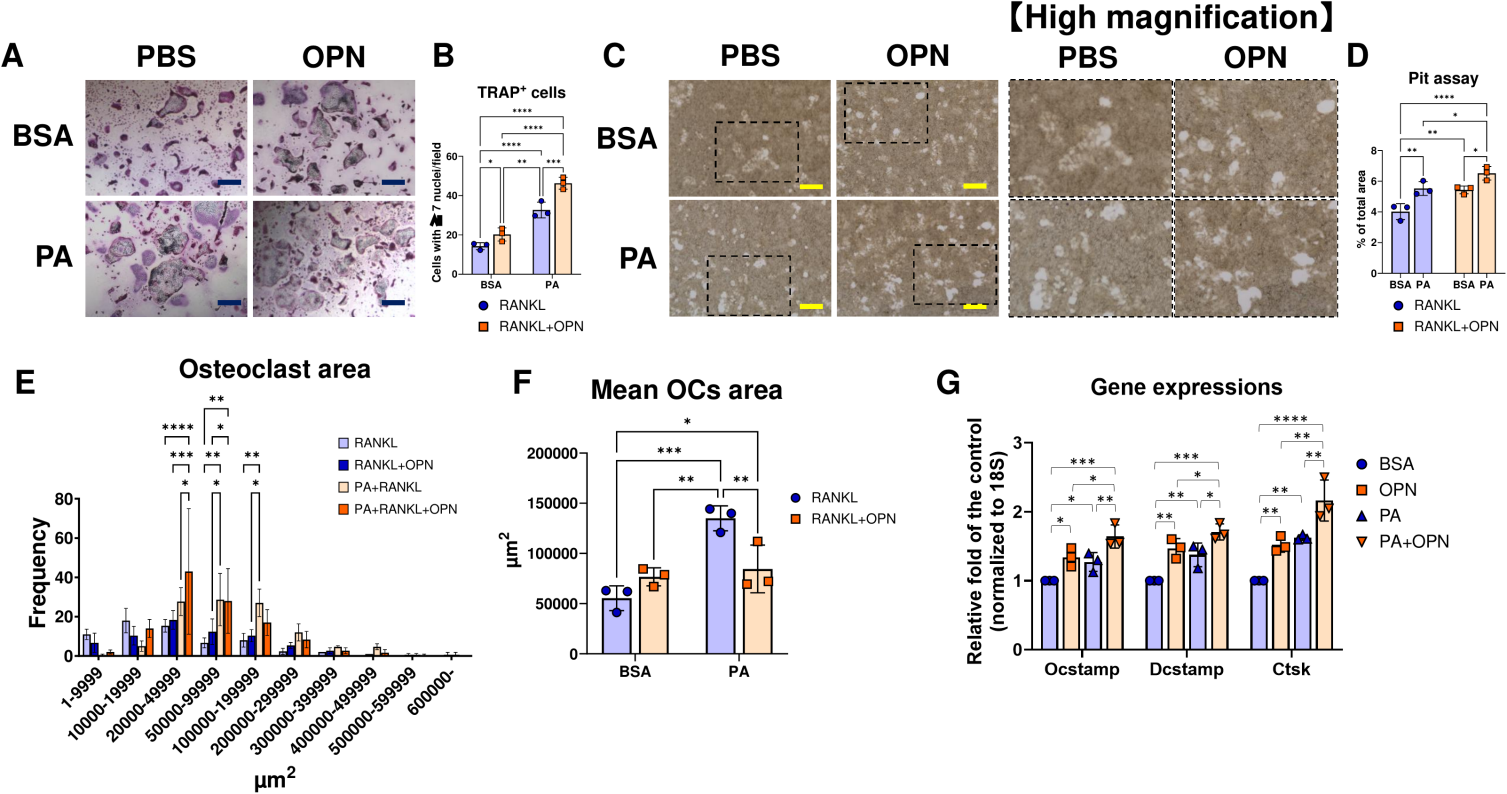
Assessment of the effect of PA priming and OPN on the osteoclastic differentiation and activity of RANKL-treated BMMs. **(A)** Representative photos of TRAP staining in RANKL-treated BMMs with or without OPN after pre-incubation with BSA or palmitate. **(B)** Plots for the quantified number of each condition. **(C)** Representative photos of pit assay of RANKL-treated BMMs with or without OPN after pre-incubation with BSA or palmitate. Magnified photos corresponding to the dashed rectangles are shown in each right image. **(D)** Plots for the ratio of pit area to the field in each condition. **(E)** Histogram of classified osteoclasts in each condition. **(F)** Mean osteoclast area in each condition. **(G)** Gene expression of the RANKL-treated BMMs under each condition. *p < 0.05, **p< 0.01, ***p< 0.001, ****p< 0.0001. All individual data are shown in a scatter plot (n = 3). Data are presented as mean ± SD. P-values were determined by two-way ANOVA followed by *post hoc* tests.

**Figure 9:**
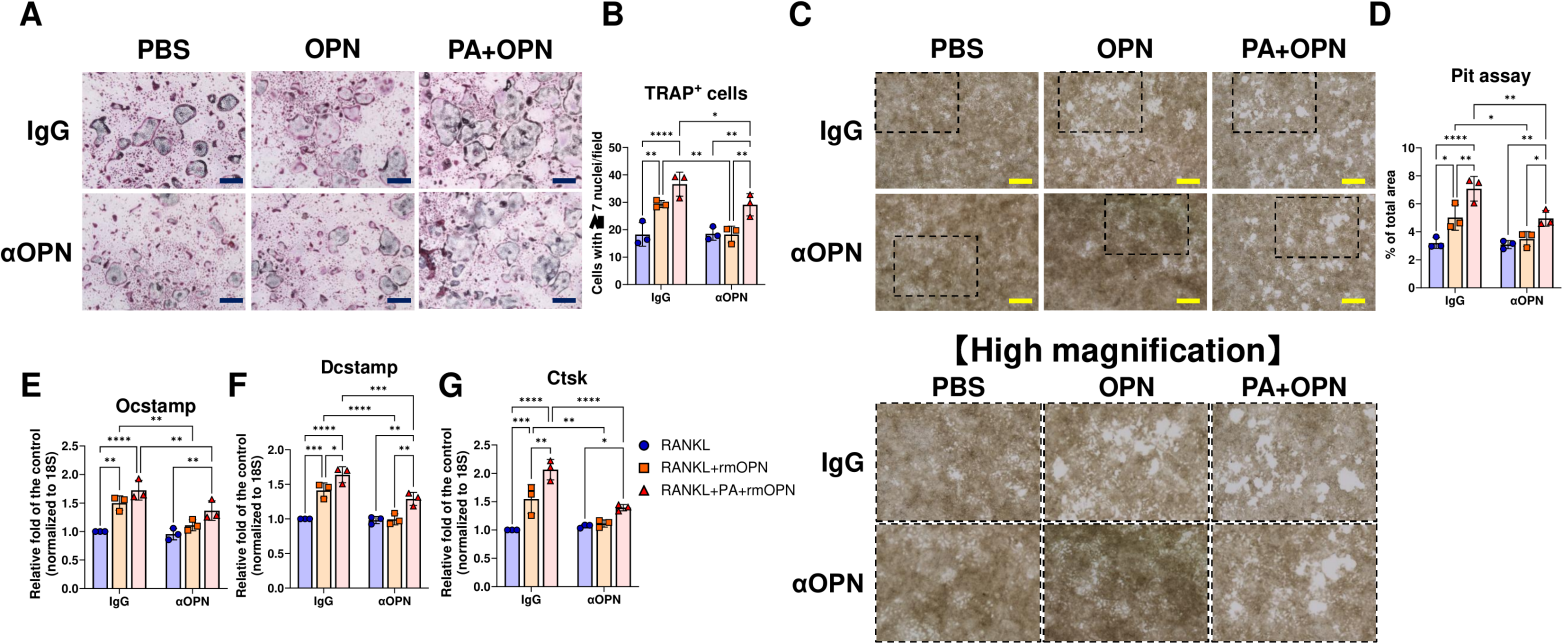
Assessment of the effect of OPN neutralizing antibody on the OPN-mediated osteoclastic differentiation and activity of RANKL-treated BMMs. **(A)** Representative photos of TRAP staining in RANKL-treated BMMs with or without OPN after pre-incubation with BSA or palmitate in the presence of OPN neutralizing antibody. **(B)** Plots for the quantified number of each condition. **(C)** Representative photos of pit assay of RANKL-treated BMMs with or without OPN after pre-incubation with BSA or palmitate in the presence of OPN neutralizing antibody. Magnified photos corresponding to the dashed rectangles are shown in each lower image. **(D)** Plots for the ratio of pit area to the field in each condition. **(E)** *Ocstamp*, **(F)** *Dcstamp*, and **(G)** *Ctsk* expression of the RANKL-treated BMMs under each condition. *p < 0.05, **p< 0.01, ***p< 0.001, ****p< 0.0001. All individual data are shown in a scatter plot (n = 3). Data are presented as mean ± SD. P-values were determined by two-way ANOVA followed by *post hoc* tests.

To investigate whether OPN contributes to the promotion of osteoclastogenesis in coordination with PA, mouse BMMs were stimulated with RANKL in the presence or absence of PA and OPN. TRAP staining revealed that both PA priming and OPN treatment enhanced osteoclastogenesis in BMMs, respectively (**Figures 8A, B**). The pit assay showed that both PA priming and OPN treatment significantly enhanced osteoclast resorptive activity (**Figures 8C, D**). Their co-stimulation further enhanced the osteoclast differentiation and activity (**Figures 8A–D**). Furthermore, PA priming significantly increased the osteoclast area in RANKL-treated BMMs (**Figure 8E**) as well as the mean osteoclast size (**Figure 8F**). In contrast, OPN significantly decreased the mean osteoclast area under PA priming, resulting from increased middle size of osteoclasts (**Figures 8E, F**). qPCR analysis revealed that both PA priming and OPN treatment upregulated osteoclastogenic markers, including *Osteoclast Stimulatory Transmembrane Protein* (*Ocstamp*), *Dendrocyte Expressed Seven Transmembrane Protein* (*Dcstamp*), and *Cathepsin K* (*Ctsk*), on day 5 after RANKL treatment (**Figure 8G**). An OPN neutralizing antibody diminished OPN-mediated osteoclast differentiation and activation in RANKL-treated BMMs in the presence or absence of palmitate pretreatment (**Figures 9A–E**).

## 4 Discussion

Several epidemiological studies have reported an association between obesity and periodontitis (37). Visceral adipose tissue plays a crucial role in the development of obesity by undergoing enlargement to store excess energy, leading to weight gain, as well as by secreting various adipokines that contribute to metabolic dysfunction and systemic micro-inflammation, which is defined as an increase in high-sensitivity C-reactive protein (hsCRP) within the healthy range and subsequently results in insulin resistance (38). CRP is produced in the liver in response to IL-6, which can be secreted by mature adipose tissue through interactions between adipocytes and immune cells, such as macrophages and T helper 17 cells (14, 39). Elevated hsCRP levels have been observed in individuals with mild obesity, which may reflect an increased prevalence of metabolic disorders (39). The progression of periodontitis may also be modified by obesity; however, whether periodontitis is exacerbated in proportion to the extent of inflammation in eWAT remains unclear.

We previously reported that adipocyte-specific CCL19-overexpressing CCL19-KI mice displayed significantly greater weight gain, glucose intolerance, and insulin resistance than those in WT mice under a 40% HFD, which resulted from advanced maturation of eWAT through enhanced maturation and immune cell infiltration in eWAT mediated by CCL19 overexpression (15). Importantly, CCL19-KI mice fed a 40% HFD exhibited significantly higher body weight than that in ND-fed CCL19-KI mice but lower body weight than that in CCL19-KI mice fed a 60% HFD. Asian populations typically exhibit glucose intolerance and insulin resistance under mild obesity (body mass index [BMI] = 23–25 kg/m^2^), whereas Western populations tend to develop these under severe obesity (BMI > 30 kg/m^2^) (40). Taken together, CCL19-KI mice fed a Western diet may be useful for studying the association between diseases and mild obesity, as their metabolic profiles resemble features of obesity observed in Asian populations with a more westernized lifestyle. Therefore, we hypothesized that CCL19-KI mice fed a 40% HFD mimic mild obesity, which is commonly observed in Asian populations and is reportedly associated with increased susceptibility to obesity-related diseases and comorbidities due to elevated micro-inflammation (40). In this study, we investigated the contribution of chronic inflammation in mature adipose tissue to the progression of experimental periodontitis under mild obesity using WT and CCL19-KI mice fed diets containing different fat contents.

In this study, we confirmed that the body weight of CCL19-KI mice was significantly higher than that of WT mice under a 40% HFD during LIP (**Figure 1B**), suggesting a sustained systemic impact, including enhanced insulin resistance and glucose intolerance, resulting from CCL19 overexpression in eWAT. μCT analyses revealed that both WT and CCL19-KI mice showed an increase in alveolar bone loss depending on the fat content of the diet, as expected (**Figures 1C–G**). Notably, LIP-induced alveolar bone loss in WT mice fed a 40% HFD was not significantly higher than that in ND-fed WT mice, whereas that in 40% CCL19-KI mice was significantly greater than that in ND-fed CCL19-KI mice (**Figures 1C–G**). Additionally, alveolar bone loss in WT mice fed a 60% HFD was significantly greater than that in WT mice fed an ND or 40% HFD, whereas alveolar bone loss in CCL19-KI mice fed a 60% HFD was significantly greater than that in ND-fed mice but comparable to that in CCL19-KI mice fed a 40% HFD (**Figures 1C–G**). Comparison of WT and CCL19-KI mice revealed that ligatured CCL19-KI mice exhibited significantly increased alveolar bone loss compared to ligatured WT mice under an ND or 40% HFD, while showing comparable alveolar bone loss to ligatured WT mice under a 60% HFD (**Figures 1C–G**). Body weight gain, glucose intolerance, and insulin resistance in CCL19-KI mice were comparable to those in WT mice fed 60% HFD, suggesting that the effect of CCL19 overexpression in eWAT was masked (15).

In CCL19-KI mice with ligatures, these inflammatory genes and *Rankl* expression in the gingiva were significantly upregulated compared to those in WT mice with ligatures under a 40% HFD, along with an increased *Rankl to Opg* ratio (**Figures 2A–F**). Consistent with qPCR results in the gingiva (**Figure 2**) and alveolar bone loss (**Figure 1**), the number of TRAP-positive cells in ligatured CCL19-KI mice was significantly higher than that in ligatured WT mice under a 40% HFD and comparable to that in WT mice under a 60% HFD (**Figure 3**). These findings suggest that periodontal inflammation and subsequent osteoclastogenesis are exacerbated in CCL19-KI mice under a 40% HFD, potentially resulting from differences in weight gain and/or enhanced metabolic abnormalities.

We also considered serum FFA as a potential factor derived from mature eWAT promoting LIP under a 40% HFD, as we previously found that CCL19-KI mice fed a 40% HFD showed significantly elevated serum FFA levels compared with WT mice fed the same diet, potentially contributing to increased glucose intolerance and insulin resistance (15). As expected, ligatured CCL19-KI mice exhibited significantly higher serum FFA levels than ligatured WT mice under a 40% HFD (**Figure 4A**). The pattern of proinflammatory cytokine expressions, such as Tnfα, Il-1β, and Mcp-1 in the eWAT was similar to serum FFA levels (**Figures 4B–D**), suggesting increased adipose inflammation in CCL19-KI mice than WT mice under a 40% HFD.

Peritoneal macrophages from CCL19-KI mice fed either an ND or a 40% HFD displayed significantly higher LPS-induced gene expression and TNF-α secretion than those from WT mice under the same diets (**Figures 5A, B**). PA, one of the predominant saturated fatty acids in the blood, can activate the NF-κB pathway via Toll-like receptor 4 (TLR4) and prime TLR4-mediated inflammatory responses, which may contribute to the pathogenesis of obesity- and diabetes-related systemic disorders (41, 42). We also confirmed that LPS-induced TNF-α expression was enhanced by both pre-stimulation (priming) and co-stimulation with PA in RAW264.7 cells (**Figures 5C–F**). In addition, we verified that rmCCL19 did not induce an inflammatory response in RAW264.7 cells at concentrations from 0.1 ng/mL (comparable to the serum CCL19 of 60% HFD-fed mice (15)) to 10 ng/mL (**Supplementary Figure 9**). These results suggest that peritoneal macrophages might be primed by elevated FFAs, most likely PA, under a 40% HFD. Further investigations utilizing clodronate-induced macrophage depletion or bone marrow transplantation between ND and HFD-fed mice, or WT and CCL19-KI mice could be helpful to prove the importance of FFA-primed macrophages in the exacerbation of LIP. In addition, determination of PA concentrations in blood and peripheral tissues (e.g., eWAT, gut, and gingiva) by lipidome analysis would provide useful data to understand the precise role of PA in the exacerbation of LIP under obesity. Stearic acid is known to induce an inflammatory response in macrophages (43, 44), whereas the role of stearic acid on osteoclastogenesis has not been investigated. It would be worthwhile to determine it in a future study. Furthermore, an *in vitro* study treating peritoneal macrophages from WT mice with serum from HFD-fed WT or CCL19-KI mice in combination with a TLR4 antagonist, to block FFA-mediated priming, to determine pro-inflammatory mediators other than PA, should be conducted in the future.

Ligatured CCL19-KI mice fed a 40% HFD showed significant upregulation of *Spp1* in eWAT (**Figures 6B–I**) and elevated serum OPN levels compared to those in ligatured WT mice fed the same diet. Notably, serum OPN levels showed a pattern consistent with LIP-induced alveolar bone loss (**Figure 6J**). OPN has been reported to play a role in bone remodeling and resorption in certain diseases. Specifically, OPN promotes differentiation of BMMs into osteoclasts, exacerbating osteoarthritis by enhancing bone turnover and remodeling (45). OPN may also link bone remodeling to increased bone metastasis in cancer (32), and a recent study suggested OPN as a novel marker and therapeutic target for breast cancer metastasis (46). OPN was mainly detected in the bilateral (root and bone) side of the periodontal ligament of the alveolar bone (**Supplementary Figure 10**), similar to the previous report (47).

Another recent study using mice with bilateral eWAT removal or macrophage depletion via clodronate liposomes demonstrated that macrophages in eWAT are the primary source of serum OPN in obesity (35). DNA microarray analysis of eWAT from lean and obese mice showed that *Spp1* is the most highly upregulated gene in mice fed an HFD for 12 weeks (48). Our DNA microarray analysis in differentiated 3T3-L1 cells co-cultured with LPS-stimulated RAW264.7 cells indicated that adipocytes can also serve as a source of SPP1, particularly in inflamed mature adipose tissue (39) (**Supplementary Figure 11**). In addition, CCL19-KI mice fed a 40% HFD exhibited increased CD11c-positive dendritic cells and F4/80-positive macrophages in eWAT, contributing to enhanced adipose inflammation (15). Consistently, we confirmed that ligatured CCL19-KI mice exhibited significantly larger F4/80 and OPN-positive area than ligatured WT mice under a 40% HFD (**Figures 7A–C**). Notably, the F4/80-positive area, indicating infiltrated M1 macrophages, in eWAT of ligatured CCL19-KI mice was dramatically increased compared to that of ligatured WT mice under a 40% HFD (**Figures 7A, B**), suggesting the maturation of eWAT of 40% HFD-fed CCL19 KI mice. Most of the OPN-positive area overlapped with the F4/80-positive area (**Figure 7D**), supporting the previous report showing macrophages as the main source of OPN in eWAT (35). Collectively, these findings suggest that serum OPN upregulation in ligatured CCL19-KI mice fed a 40% HFD reflects accelerated visceral adipose tissue inflammation via enhanced interactions between adipocytes and immune cells.

Clinical studies have reported that body weight is negatively correlated with bone mass when the confounding effect of gravity is excluded (49, 50). In this study, LIP was initiated in mice after 12 weeks of either a 40% or 60% HFD, a time point at which *Spp1* upregulation in eWAT was observed. OPN regulates matrix mineralization by stabilizing mineral precursor phases and binding to crystal surfaces (51), and it also mediates cell attachment of osteocytes and osteoclasts (52–55). In addition, OPN facilitates macrophage infiltration into adipose tissue in HFD-fed mice by promoting macrophage recruitment (56–58). OPN exerts its biological functions through interactions with integrins and CD44, thereby participating in the regulation of inflammation, immune responses, and bone metabolism (59). OPN has been reported to exhibit dual regulatory effects on osteoclastogenesis: it promotes osteoclastic differentiation and activity in mouse BMMs treated with RANKL (45), while also inhibiting osteoclastogenesis in these cells via lactate accumulation as negative feedback (35). In this study, rmOPN at 100 ng/mL—corresponding to the difference in mean serum OPN levels between WT and CCL19-KI mice—significantly promoted osteoclastogenesis in RANKL-treated mouse BMMs (**Figures 8A, B**). Pit assays further revealed that OPN promoted osteoclast activity in mouse BMMs (**Figures 8C, D**).

We found that PA priming exerted a promotive effect on osteoclastogenesis (**Figures 8A, B**) and significantly enhanced osteoclast activity (**Figures 8C, D**), in agreement with previous studies showing that co-stimulation with PA promotes osteoclastogenesis in BMMs (60, 61). Consistent with this elevated activity, RANKL-treated BMMs primed with PA displayed a significant increase in larger osteoclasts compared to those in RANKL-treated BMMs without PA exposure (**Figures 8E, F**). Furthermore, we found that treatment of OPN significantly increased osteoclast activity in RANKL-treated BMMs primed with PA (**Figures 8C, D, G**), despite a reduced mean area of differentiated osteoclasts (**Figure 8F**). This apparent discrepancy could be explained by the increase in the middle size of osteoclasts by PA-priming and OPN (**Figure 8**). OPN neutralizing antibody successfully suppressed the OPN’s effects on the RANKL-treated BMMs but did not interfere with the effect of PA on them (**Figures 9A–E**). OPN can exert both anti- and pro-apoptotic effects on osteoclast precursors depending on the pathogenic condition (62, 63).

The extent of alveolar bone loss of 40% HFD-fed CCL19-KI mice was competitive with 60% HFD-fed both WT and CCL19-KI mice with ligatures (there was no significant difference in alveolar bone loss between WT and CCL19-KI mice under 60% HFD) despite the difference in body weight (**Figures 1B–G**). Serum FFA (**Figure 4A**) and OPN levels (**Figure 6J**), as well as adipose inflammation, such as gene expressions (**Figures 4B–D**), and macrophage infiltration (**Figures 7A, B**) of 40% HFD-fed ligatured CCL19-KI mice were also competitive with 60% HFD-fed both WT and CCL19-KI mice with ligatures. These data suggested that the maturation of eWAT in 40% HFD-fed CCL19-KI mice reached a ‘plateau’ similar to 60% HFD-fed WT and CCL19-KI mice. To the best of our knowledge, this is the first study demonstrating OPN derived from eWAT contributes to the exacerbation of periodontitis in cooperation with elevated FFA levels under mild obesity. Improving circulating FFA levels and targeting circulating OPN using approaches such as neutralizing antibodies or receptor inhibitors may represent effective therapeutic strategies for preventing the progression of periodontitis under mild obesity. Additionally, it can be conceivable that other circulating inflammatory mediators, rather than OPN and PA, derived from eWAT in accordance with obesity, and possibly epigenetic changes in myeloid cell progenitors, may be involved in the molecular basis of the findings in the current study. Further investigations to unveil them are necessary.

This study has certain limitations. First, interventional studies using either OPN-neutralizing antibodies, fatty acid synthesis inhibitors, or genetic ablation of *Spp1* in macrophages and/or adipocytes are required to confirm the findings, especially the relationship between increased serum OPN and enhanced alveolar bone loss. Second, only male mice were used in the *in vivo* experiments to avoid potential effects of the estrous cycle on LIP. Future studies should include female mice to determine whether similar results are observed. Third, further investigations are needed to provide more convincing evidence elucidating the molecular mechanisms by which PA and OPN promote osteoclastic differentiation and activity. Fourth, studies using adipocyte-specific Ccl19-null mice would be a suitable application to prove the importance of the CCL19-CCR7 axis in the adipose tissue inflammation from the viewpoint of loss-of-function. However, adipocyte-specific homologous recombination was challenging since there are 11 pseudogenes located near murine Ccl19 (15). Alternatively, CCR7 antagonism by CCR7 neutralizing antibody or specific inhibitor in HFD-fed mice should be conducted in future studies. Lastly, no clinical study in Asian to support the association of serum/adipose CCL19 levels with the degree of obesity and maturation of adipose tissue has been conducted whereas a clinical study in middle East country has reported that CCL19 gene expression in subcutaneous adipose tissue (SAT) was correlated with BMI, HOMA-IR, serum CRP, and proinflammatory and chemokine expression in SAT (64). A clinical study in the Asian population to demonstrate the association of serum and adipose CCL19 levels with the degree of obesity and maturation of adipose tissue will be required in the future.

## 5 Conclusions

**Figure 10:**
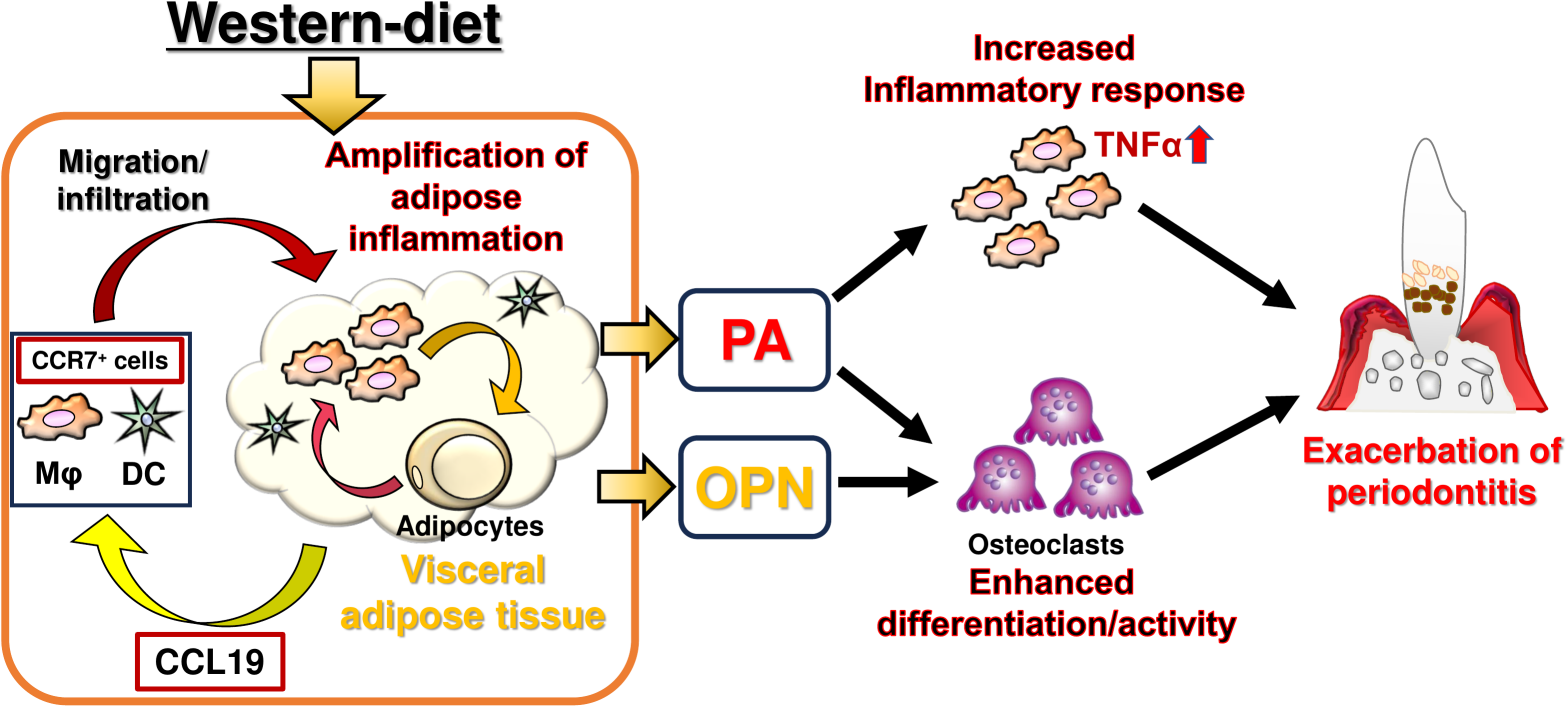
Summary of the current study. DCs, dendritic cells; Mφ, macrophages; PA, palmitic acid; OPN, osteopontin.

In summary, metabolically abnormal visceral adipose tissue leads to elevated circulating FFAs and upregulated OPN/*Spp1*, thereby contributing to the exacerbation of periodontitis through enhanced inflammation in macrophages and osteoclastic differentiation and activity in mild obesity (**Figure 10**). Future studies should be required to demonstrate the contribution of FFA-priming in macrophages and circulating OPN in the exacerbation of periodontitis by *in vivo* interventional approaches.

## Data Availability

All data published in the current article are available upon reasonable request. The RNA-sequencing dataset was uploaded to the NCBI GEO repository (GSE328134).
