# Supplementary figures and images for: Adipose tissue inflammation mediated by CCL19 overexpression exacerbates experimental periodontitis via elevated circulating saturated fatty acids and osteopontin in Western-diet-fed mice
Source: Front Immunol. 2026 May 1;17:1787572. doi: 10.3389/fimmu.2026.1787572 (PMC13176198; doi:10.3389/fimmu.2026.1787572)

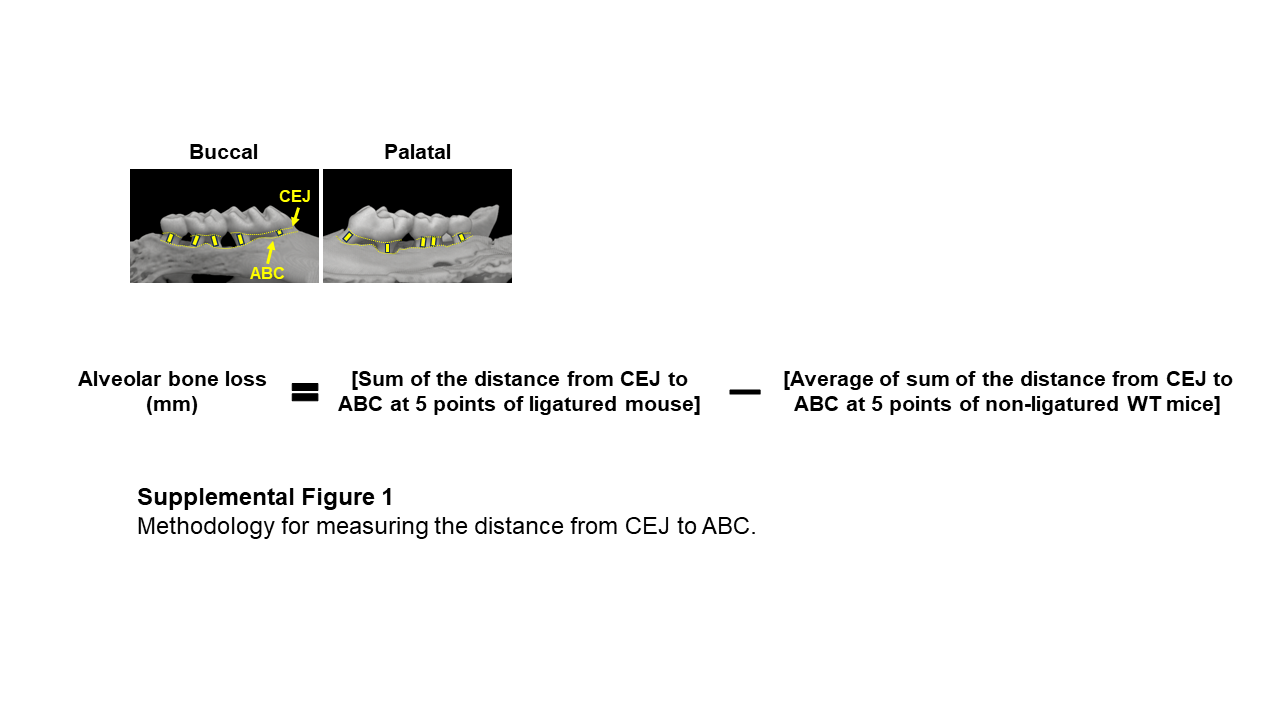

Supplement: Supplementary file 1 [file Image1.tif]

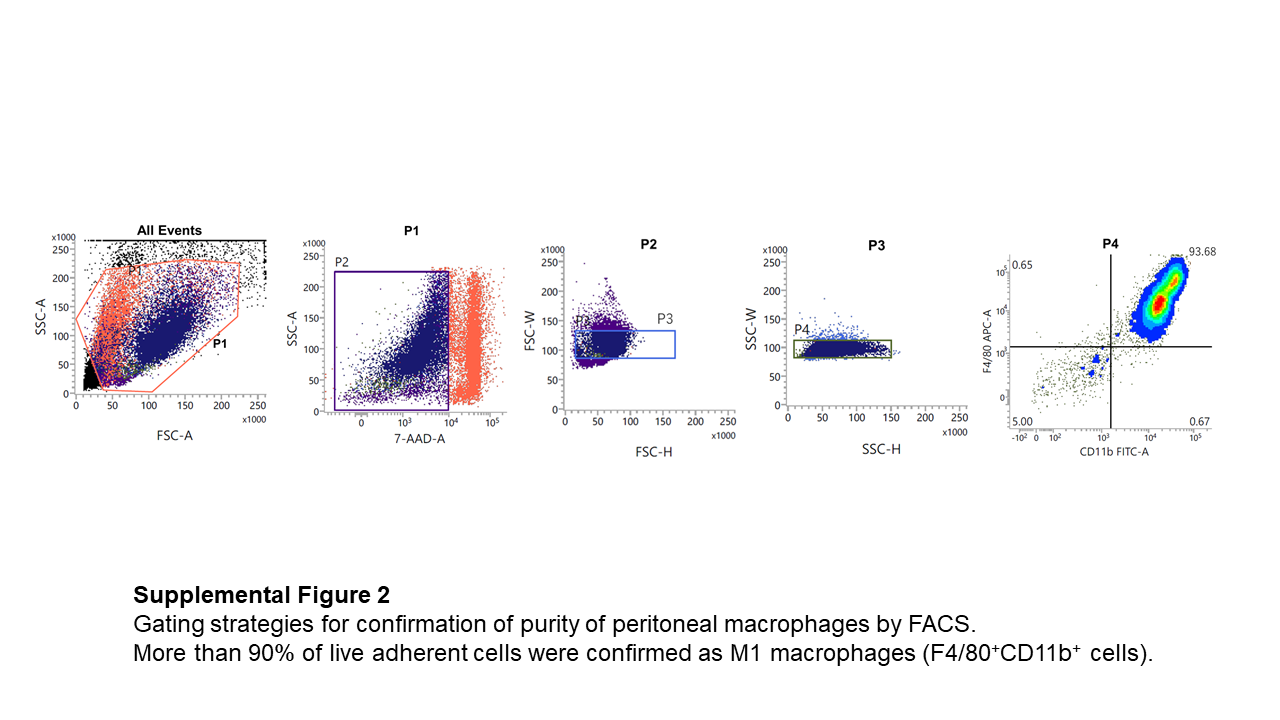

Supplement: Supplementary file 2 [file Image2.tif]

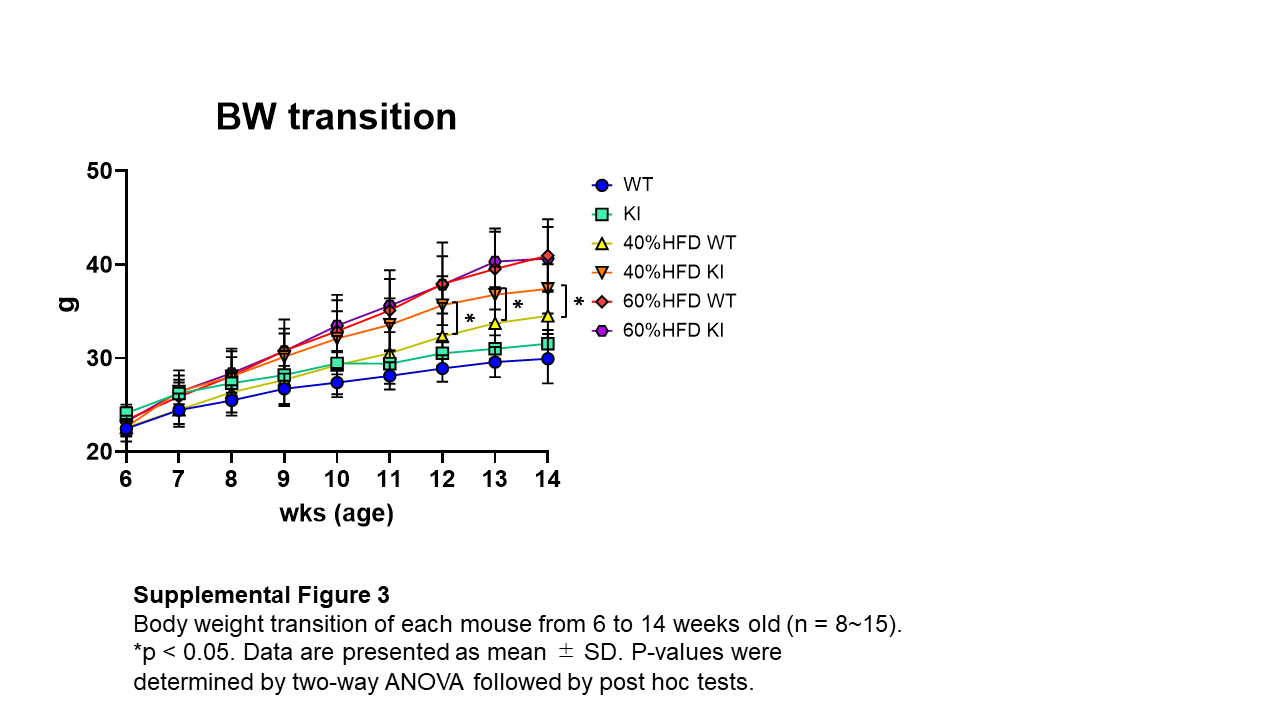

Supplement: Supplementary file 3 [file Image3.tif]

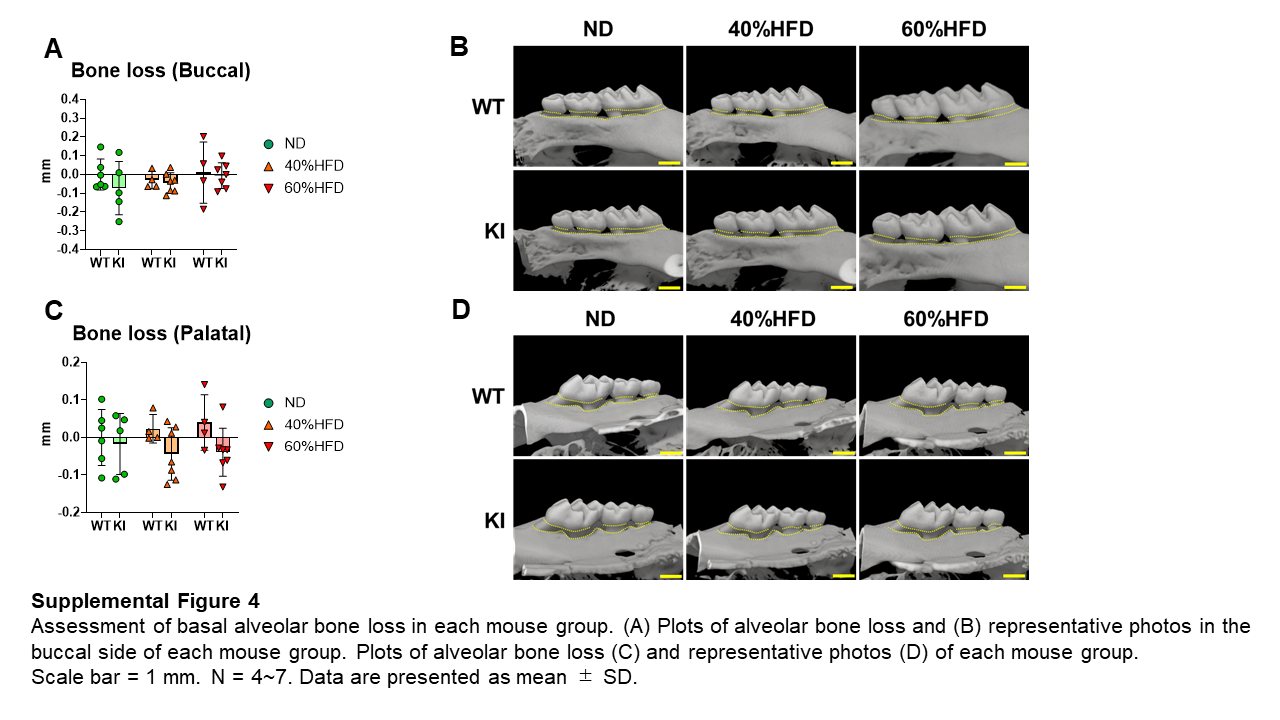

Supplement: Supplementary file 4 [file Image4.tif]

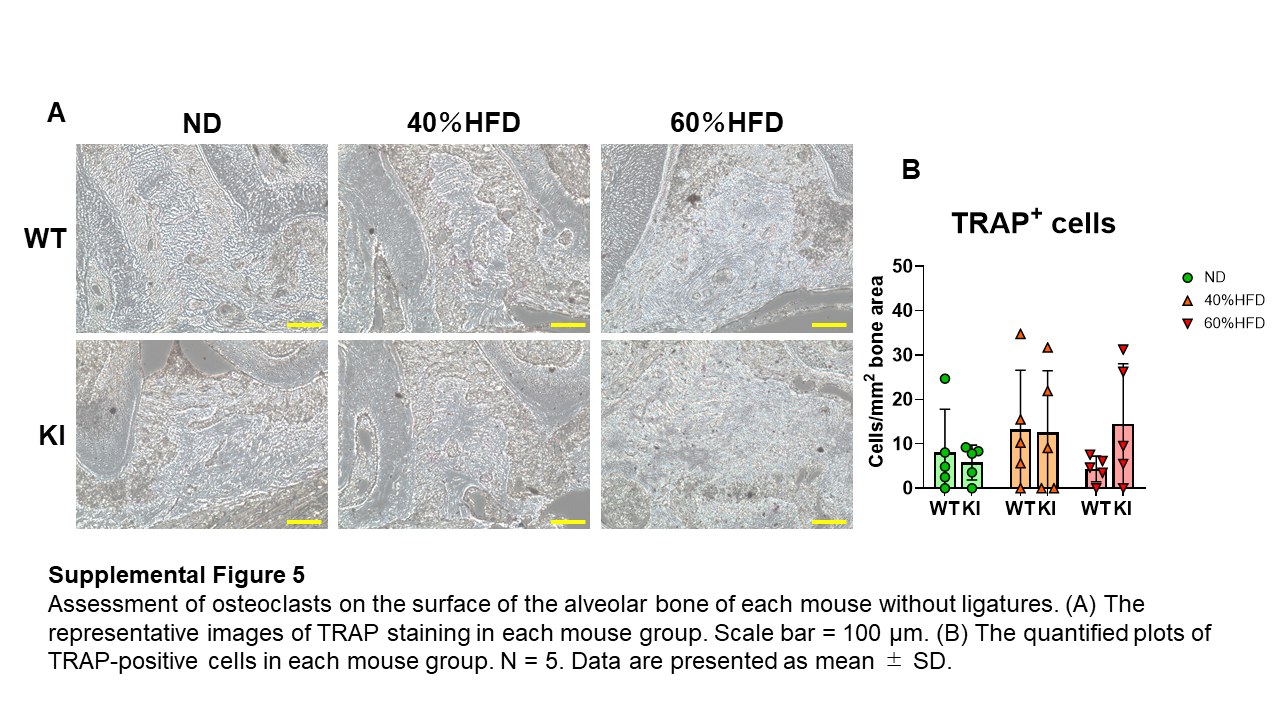

Supplement: Supplementary file 5 [file Image5.tif]

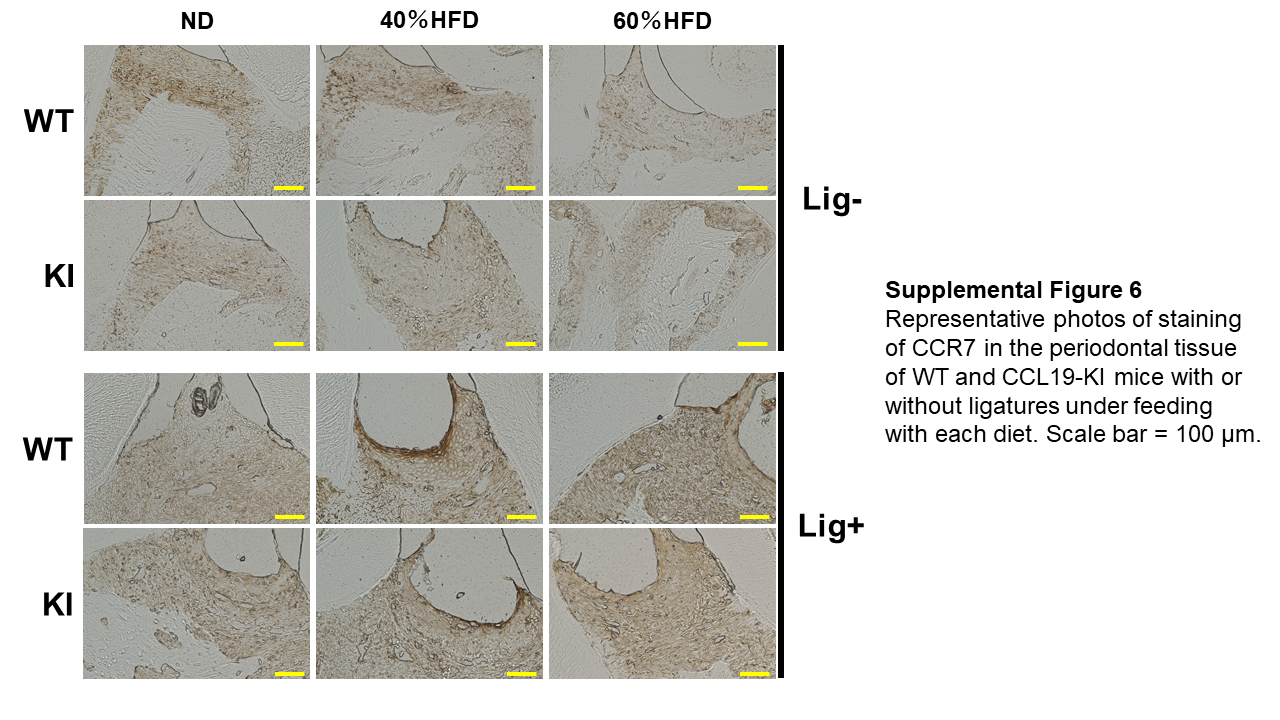

Supplement: Supplementary file 6 [file Image6.tif]

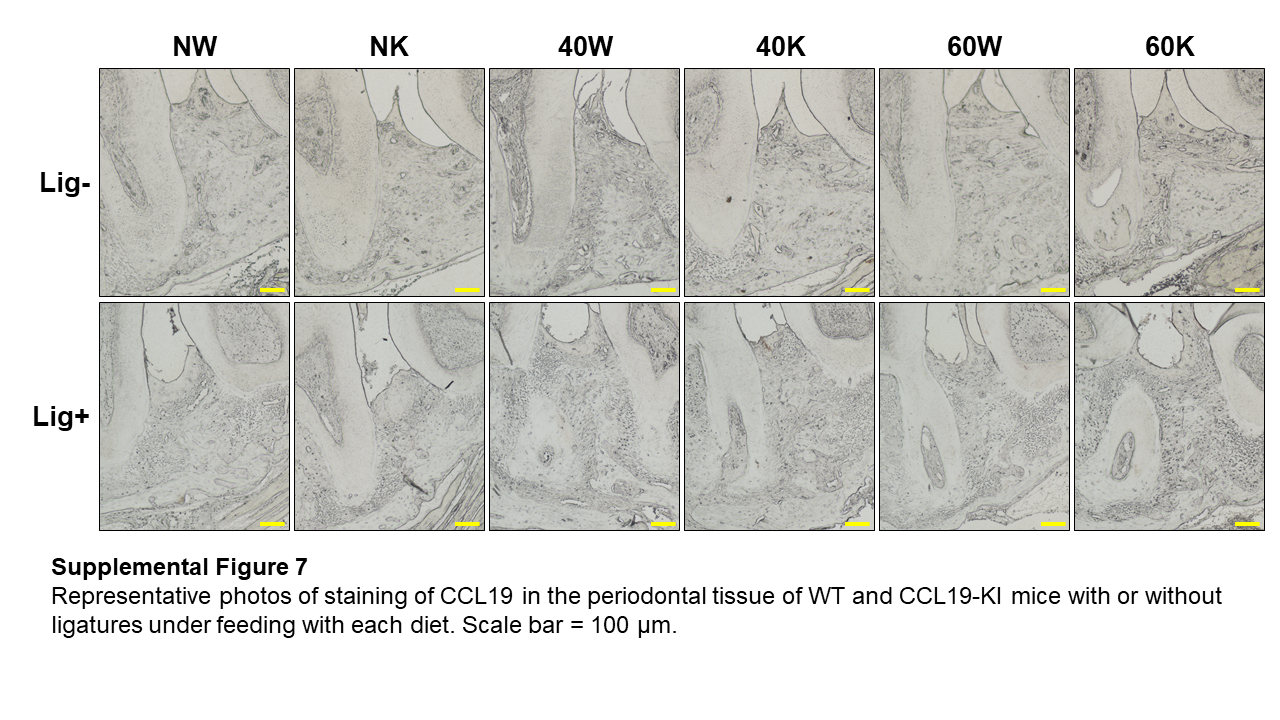

Supplement: Supplementary file 7 [file Image7.tif]

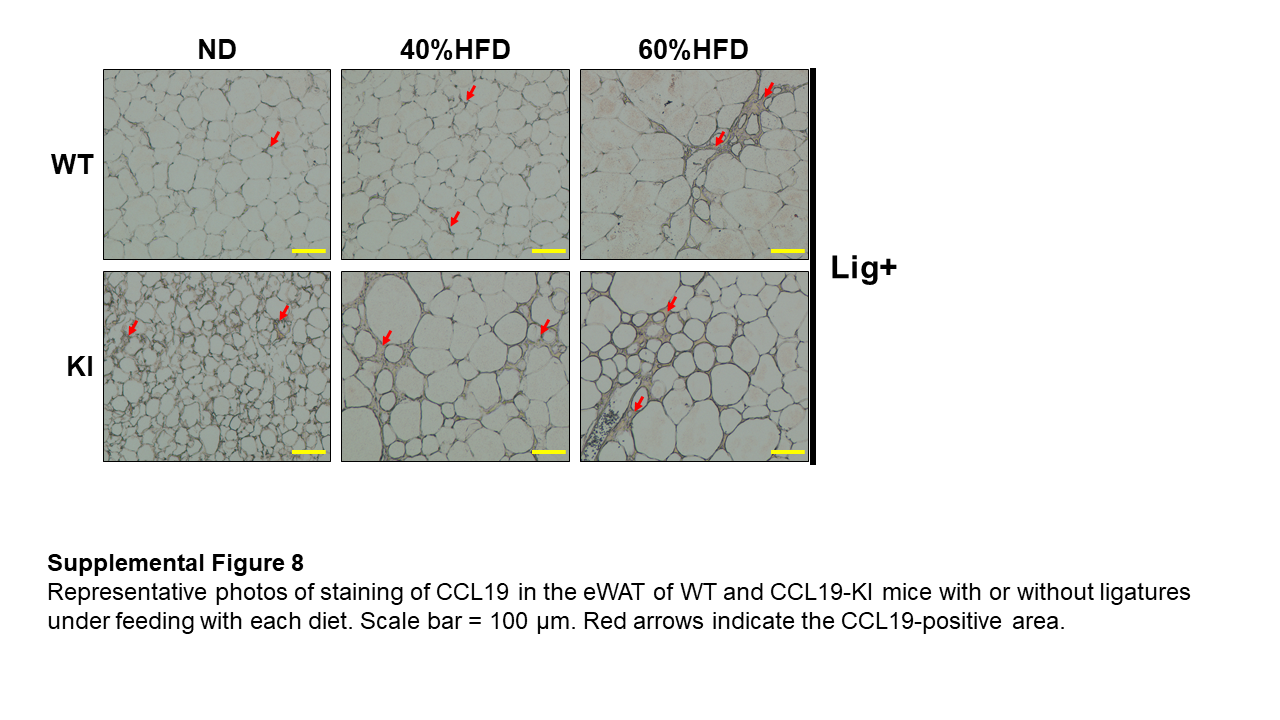

Supplement: Supplementary file 8 [file Image8.tif]

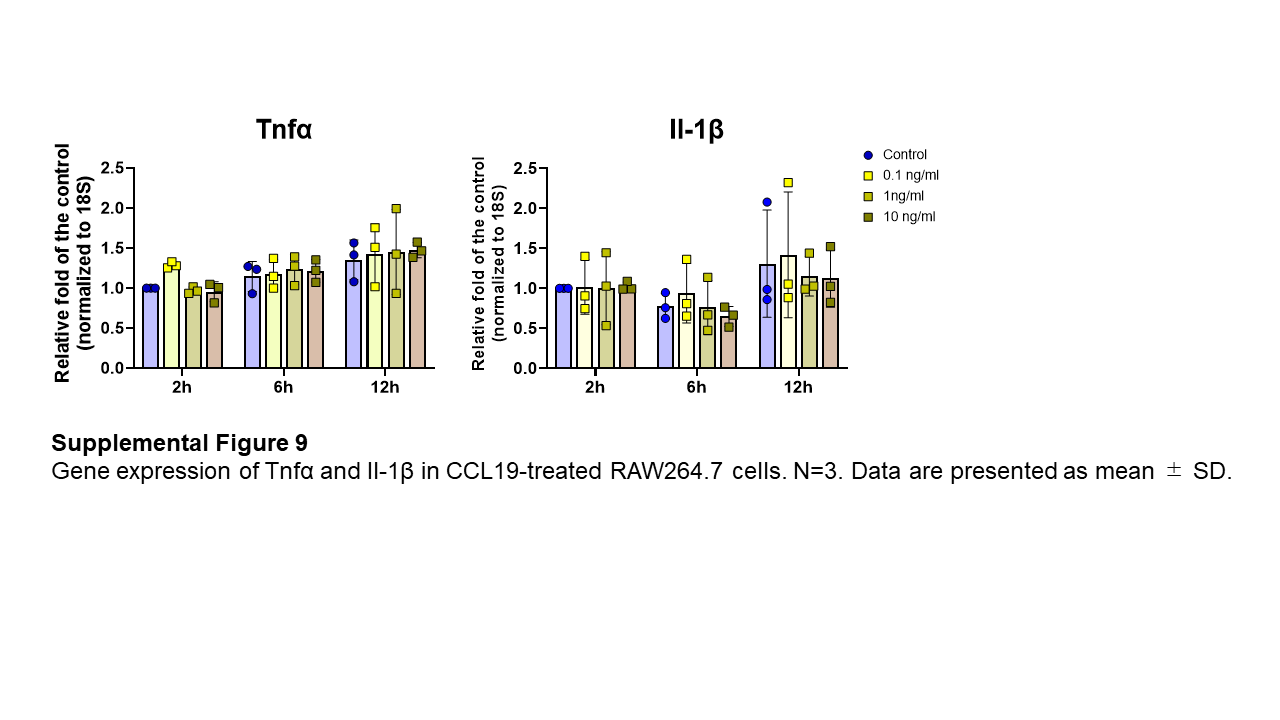

Supplement: Supplementary file 9 [file Image9.tif]

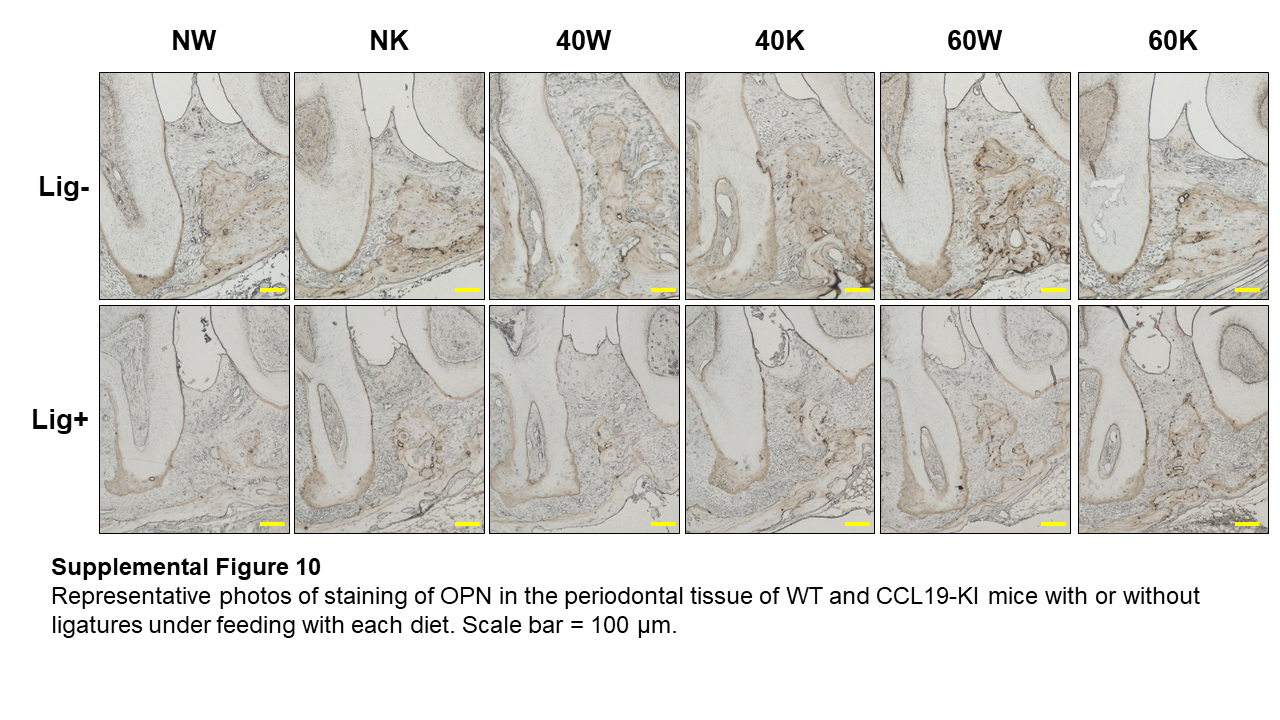

Supplement: Supplementary file 10 [file Image10.tif]

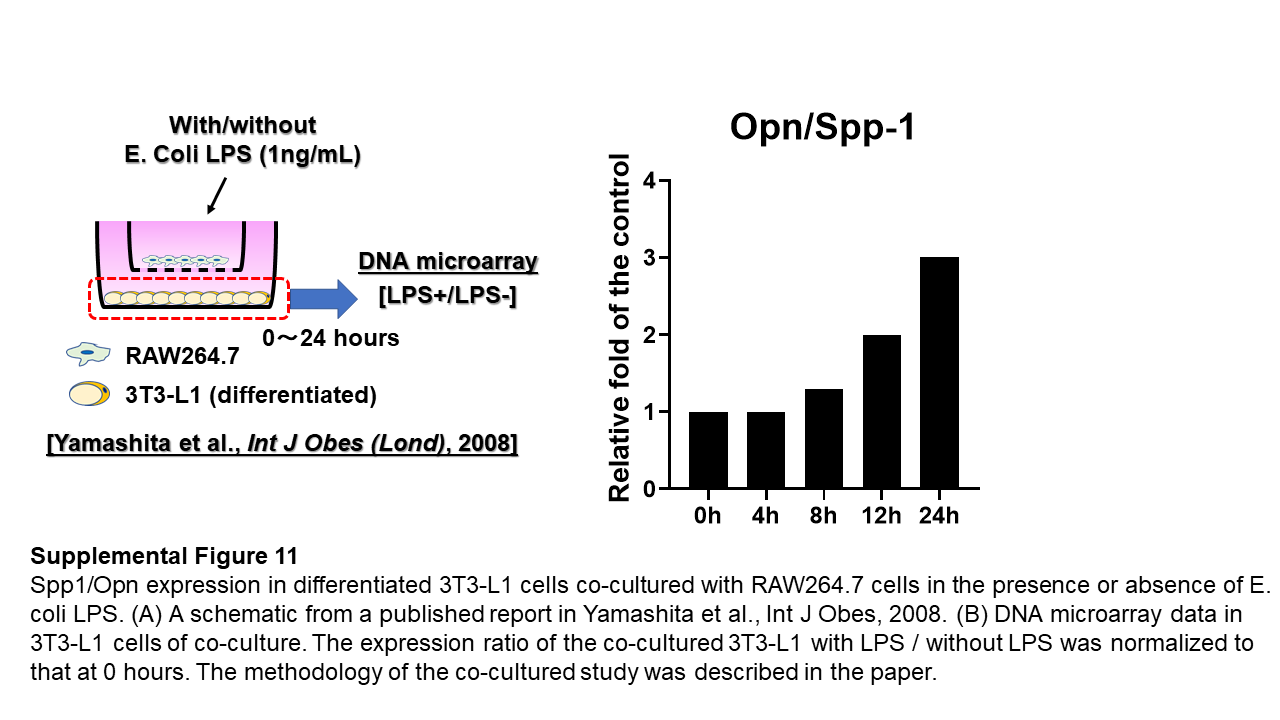

Supplement: Supplementary file 11 [file Image11.tif]
