# Supplementary material for: Adipose tissue inflammation mediated by CCL19 overexpression exacerbates experimental periodontitis via elevated circulating saturated fatty acids and osteopontin in Western-diet-fed mice
Source: Front Immunol. 2026 May 1;17:1787572. doi: 10.3389/fimmu.2026.1787572 (PMC13176198; doi:10.3389/fimmu.2026.1787572)
Supplement: Supplementary file 12 [file Table1.docx]

Supplemental Table 1. Primer sequences

| Gene | Sequence |
| --- | --- |
| mTnfα forward | GACAGTGACCTGGACTGTGG |
| mTnfα reverse | TGAGACAGAGGCAACCTGAC |
| mIl-1b forward | GAAGAAGAGCCCATCCTCTG |
| mIl-1b reverse | TCATCTCGGAGCCTGTAGTG |
| mIl-17a forward | AAGGCAGCAGCGATCATCC |
| mIl-17a reverse | GGAACGGTTGAGGTAGTCTGAG |
| mRankl forward | CGCTCTGTTCCTGTACTTTCG |
| mRankl reverse | GAGTCCTGCAAATCTGCGTT |
| mOpg forward | CCTTGCCCTGACCACTCTTAT |
| mOpg reverse | CACACACTCGGTTGTGGGT |
| mSpp1 (Opn) forward | GCTTGGCTTATGGACTGAGGTC |
| mSpp1 (Opn) reverse | CCTTAGACTCACCGCTCTTCATG |
| mDc-stamp forward | AAAACCCTTGGGCTGTTCTT |
| mDc-stamp reverse | AATCATGGACGACTCCTTGG |
| mOc-stamp forward | GGCTCAGAAGTTACCCACTGTC |
| mOc-stamp reverse | GGAGGTTGGTTGAGGACGAAGA |
| mCtsk forward | AATACCTCCCTCTCGATCCTACA |
| mCtsk reverse | TGGTTCTTGACTGGAGTAACGTA |
| m18S rRNA forward | GCTTAATTTGACTCAACACGGGA |
| m18S rRNA reverse | AGCTATCAATCTGTCAATCCTGTC |

All of the primers were purchased from FASMAC (Kanagawa, Japan).
