# Supplementary material for: Adipose tissue inflammation mediated by CCL19 overexpression exacerbates experimental periodontitis via elevated circulating saturated fatty acids and osteopontin in Western-diet-fed mice
Source: Front Immunol. 2026 May 1;17:1787572. doi: 10.3389/fimmu.2026.1787572 (PMC13176198; doi:10.3389/fimmu.2026.1787572)
Supplement: Supplementary file 13 [file Table2.docx]

Supplemental Table 2. Antibodies and reagents for flow cytometric staining assays.

|  | Antibody/reagent | Fluorochrome | Clone | Cat. No | Supplier |
| --- | --- | --- | --- | --- | --- |
| Anti-mouse | F4/80 | APC | BM8 | 123115 | Biolegend |
| Anti-mouse/human | CD11b | FITC | M1/70 | 101205 | Biolegend |
| Anti-mouse | CD16/32 | - | S17011E | 156604 | Biolegend |
|  | 7-AAD | - | - | 420403 | Biolegend |
